# Short‐chain mono‐carboxylates as negative modulators of allosteric transitions in *Gloeobacter violaceus* ligand‐gated ion channel, and impact of a pre‐β5 strand (Loop Ω) double mutation on crotonate, not butyrate effect

**DOI:** 10.14814/phy2.15916

**Published:** 2024-02-11

**Authors:** Catherine Van Renterghem, Ákos Nemecz, Karima Medjebeur, Pierre‐Jean Corringer

**Affiliations:** ^1^ Channel‐Receptors Unit, Institut Pasteur, CNRS UMR3571 Université Paris Cité Paris France

**Keywords:** GLIC, Loop Ω, orthotopic site, pLGIC, pre‐β5 strand, vestibular site

## Abstract

Using the bacterial proton‐activated pentameric receptor‐channel *Gloeobacter violaceus* ligand‐gated ion channel (GLIC): (1) We characterize saturated, mono‐carboxylates as negative modulators of GLIC (as previously shown for crotonate; Alqazzaz et al., *Biochemistry*, 2016, 55, 5947). Butyrate and crotonate have indistinguishable properties regarding negative modulation of wt GLIC. (2) We identify a locus in the pre‐β5 strand (Loop Ω) whose mutation inverses the effect of the mono‐carboxylate crotonate from negative to positive modulation of the allosteric transitions, suggesting an involvement of the pre‐β5 strand in coupling the extracellular orthotopic receptor to pore gating. (3) As an extension to the previously proposed “in series” mechanism, we suggest that a orthotopic/orthosteric site—vestibular site—Loop Ω—β5‐β6 “sandwich”—Pro‐Loop/Cys‐Loop series may be an essential component of orthotopic/orthosteric compound‐elicited gating control in this pentameric ligand‐gated ion channel, on top of which compounds targeting the vestibular site may provide modulation.

## Graphical abstract

Using the bacterial pentameric ligand‐gated ion channel *Gloeobacter violaceus* ligand‐gated ion channel, we show that a pair of mutations in Loop Ω (= pre‐β5 strand) inverses the pharmacological effect of crotonate, a ligand of the orthotopic site (homologous to the neurotransmitter binding site). We propose a “in series” mechanism with orthotopic site—vestibular site—Loop Ω controlling the Pro‐Loop/Cys Loop and gating.

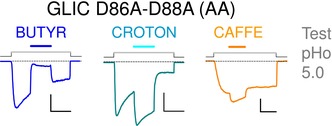

## 1 INTRODUCTION

In human body, most pentameric ligand‐gated ion channels (pLGICs) get activated by binding of neurotransmitters (acetylcholine, serotonin, GABA, glycine) to a major binding site, involved in physiological agonist effects. This pLGIC reference binding site is located in the extracellular domain (ECD), at the interface between neighbor subunits, and it is accessible from the periphery of the pentamer. This reference binding site is usually called the *orthosteric* site, but in the present report it is called the *orthotopic* site (see Section 2.2). It is also called *inter‐subunit* (inter‐SU) pocket in crystallographic studies, as well as in our mutational analysis referred to these structures. Many substances (toxins, toxic alcaloids, and chemistry products) are pharmacologically active on Eukaryote pLGICs (among which pesticides, convulsivants, drugs of abuse, and major clinically relevant substances). For many of them, the binding site is located in the transmembrane domain (TMD): either between TMD helices (propofol and several general anesthetics, alcohols, barbiturates, etc.), or within the pore lumen (picrotoxinin, pumiliotoxin, ivermectine, lindane, etc.). Regarding agents active through the ECD, they very generally bind to the reference binding site (at the interface between subunits): either to the main orthotopic agonist sites (nicotine, alpha‐bungarotoxin, etc.), or to an accessory orthotopic site involved in modulation in some heteromeric pLGICs (benzodiazepines on GABA_A_ receptors).

A new vestibular binding pocket was identified by crystallography in the ECD of several Prokaryote pLGICs. The ECD *vestibular* pocket is also called *intra‐subunit* (intra‐SU) pocket in crystallographic studies, as well as in our mutational analysis referred to these structures. The vestibular pocket is adjacent to the orthotopic pocket, but it is located within a subunit, between the ECD beta strands. It is accessible from the extracellular vestibule lumen, along the axis of the pentamer. The vestibular pocket was identified in ELIC (Spurny et al., 2012), GLIC (Fourati et al., 2015, 2020; Sauguet et al., 2013; see also acetate in Nury et al., 2010), and sTeLIC (Hu et al., 2018). The question arises whether a homologous vestibular site may become a functional drug target in Eukaryote pLGICs, a question addressed by several authors (Brams et al., 2020; Hu et al., 2018).

Here, we take advantage of the pLGIC GLIC, very well characterized regarding structures, to analyze how the vestibular site may be involved in compound‐elicited modulation of channel gating. In previously published GLIC‐carboxylates co‐crystal structures (Fourati et al., 2020; Sauguet et al., 2013), each carboxylic acid/carboxylate (CBX) compound tested was present in the co‐crystal inter‐SU (orthotopic) pocket. Some of them were in addition identified in the intra‐SU (= vestibular) pocket (see Table 1), and no compound was found elsewhere than in the two pockets, therefore referred to as CBX‐binding pockets. Van Renterghem et al. (2023) showed that integrity of the vestibular pocket is required for the modulation occurring by binding to GLIC inter‐SU (orthotopic) site. Positive modulation by di‐carboxylic acid/carboxylate (di‐CBX) compounds (fumarate, succinate), and negative modulation by caffeate, showed an “all‐or‐none” pattern of residue dependency: alanine replacement of a single residue in the inter‐SU pocket (orthotopic site), or in the intra‐SU pocket (vestibular site), abolished compound‐elicited modulation. Although di‐CBX compounds produce a positive modulation of low‐pH elicited GLIC currents, they have no agonist property.

**TABLE 1:** Mono‐CBX and di‐CBX compounds: names, structures, acidity constants, and presence in GLIC ECD carboxylate‐binding pockets in published co‐crystal structures.

Compound	Structure	Reference‐nomenclature	p*K*a	Inter‐SU pocket	Intra‐SU pocket	Pdb ref.	Ref.
Acetic acid acet	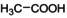	Ethanoic acid	4.76	✓	✓	3eam 4hfi^a^	Bocquet et al. (2009) and Sauguet et al. (2013)^a^
Propionic ac. propion	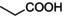	Propanoic acid	4.89	✓	✓	6hpp	Fourati et al. (2020)
Butyric acid butyr	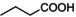	Butanoic acid	4.81	–	–	–	–
Crotonic acid croton	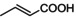	Butenoic acid	4.69	✓	0	6hji	Fourati et al. (2020)
Valeric acid valer	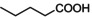	Pentanoic acid	4.82	–	–	–	–
Malonic acid malon	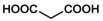	Propanedioic acid	2.85 5.70	✓	0	6hjb	Fourati et al. (2020)
Succinic acid succin	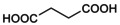	Butanedioic acid	4.21 5.63	✓	✓	6hjz	Fourati et al. (2020)
Fumaric acid fumar	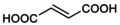	(*E*)‐but‐2‐enedioic acid	3.03 4.44	✓	0	6hj3	Fourati et al. (2020)
Glutaric acid glutar	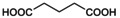	Pentanedioic acid	4.3405.42	✓	0	6hja	Fourati et al. (2020)

*Note*: Acidity constants *K*a (given as p*K*a = −log(*K*a)) were obtained (for 10 mM at 25°C, with 170 mM NaCl) from the free software Dozzzaqueux (Jean‐Marie Biansan, http://jeanmarie.biansan.free.fr/dozzzaqueux.html, in French).

^a^ Among other references.

In the present report, we use crotonic acid/crotonate (croton), previously identified as a GLIC inhibitor acting via GLIC ECD (Alqazzaz et al., 2016), and identify saturated, short‐chain mono‐CBX compounds (see Table 1) as negative modulators of the allosteric transitions (NAMs) in GLIC. We establish their “loose pattern” of residue dependency regarding the two CBX‐binding pockets. Alanine substitution of Arg77, at the border between the two pockets, has a major impact on the ability of mono‐CBXs to negatively modulate GLIC. Other substitutions in the CBX‐binding pockets have either no impact or a relatively weak impact. Within the hypothesis of the “in series” mechanism, the “loose” impact pattern leads us to propose that a (secondary) binding to the vestibular site may as well mediate negative modulation (at least with a mutated orthotopic site), and putatively modulate the influence of (primary) compound binding to the orthotopic site.

In addition, we show that a double mutation in the pre‐β5 strand, the pLGICs Loop Ω in the extracellular vestibule lumen, defined by Hu et al. (2018), inverses the effect of crotonate, from a strong negative modulation, into a strong positive modulation of pH 5 elicited currents (but without conferring agonist properties). This inversion occurs also with caffeate, but does not occur with butyrate, devoid of the double bond present in crotonate and caffeate. Although negative modulation by crotonate (on wt GLIC) shows a “loose pattern” of residue dependency, in contrast, positive modulation by crotonate (on the pre‐β5 variant) shows an “all‐or‐none” pattern of residue dependency. From our electrophysiology data, and previously published crystallographic data, we conclude that binding at the orthotopic site allows, through the vestibular region, and through the release of an Arg77‐Asp88 inter‐SU ion bridge, a pre‐β5 strand motion involved in pore gating.

## 2 MATERIALS AND METHODS

### 2.1 Electrophysiology

Electrophysiological methods were updated from Van Renterghem and Lazdunski (1994) and mostly as in Van Renterghem et al. (2023), with heterologous expression, and using an RK‐400 patch‐clamp amplifier (or a two‐electrode oocyte voltage‐clamp amplifier), pClamp and a Digidata 1500 interface for control/acquisition, and Sigmaplot 11 for data analysis and figures. We give here the main points and some differences.

#### 2.1.1 GLIC expression in tk‐ts13 cells and *Xenopus laevis* oocytes

Proton activation curves (Figure 6b) were established using as host cells commercially available (Ecocyte, Germany), ready to use, defolliculated stage 5–6 (Dumont, 1972) *Xenopus laevis* oocytes (which are unovulated, fully grown primary oocytes, *that is* at meiosis prophase 1). Surgery from the ovary, followed by removal of inner ovarian epithelium, and thecal and follicular cells, were done by the company. Such oocytes are covered only by their vitelline envelope, a protein network over the plasma membrane microvilli, which keeps them spherical. Nuclear injection of DNA was done on the day of reception of oocytes. Other electrophysiological data were obtained using whole‐cell patch‐clamp recording from tk‐ts13 host cells, devoid of endogenous Acid Sensing Ion Channels (Van Renterghem et al., 2023), and here cultured without antibiotics. Transfection was done within 24 h after cell seeding, using the DNA‐calcium phosphate co‐precipitation method. GFP‐positive cells were used for electrophysiology 1–2 days after transfection (tk‐ts13 cells), or 1–3 days after nuclear injection (oocytes). A mixture of DNAs coding for GLIC and GFP in separate pMT3 vectors was used, with amounts of [2 + 0.2] μg per 35 mm dish for transfections, or [0.04 + 0.02] g/L in water for injections. GLIC residue numbering follows Protein Data Bank (PDB) entry 3EAM (Bocquet et al., 2009), with its one residue up‐shifted numbering.

#### 2.1.2 Preparation of solutions

All solutions were prepared from the acid forms of the buffers/acido‐basic compounds (BAPTA, HEPES, MES, CBX, etc.). Stock 100 mmol/L (mM) mono‐CBX water solutions were prepared directly at pH 5 (NaOH/HCl), aliquoted and kept at −20°C.

#### 2.1.3 Whole‐cell patch‐clamp recording

**FIGURE 1:**
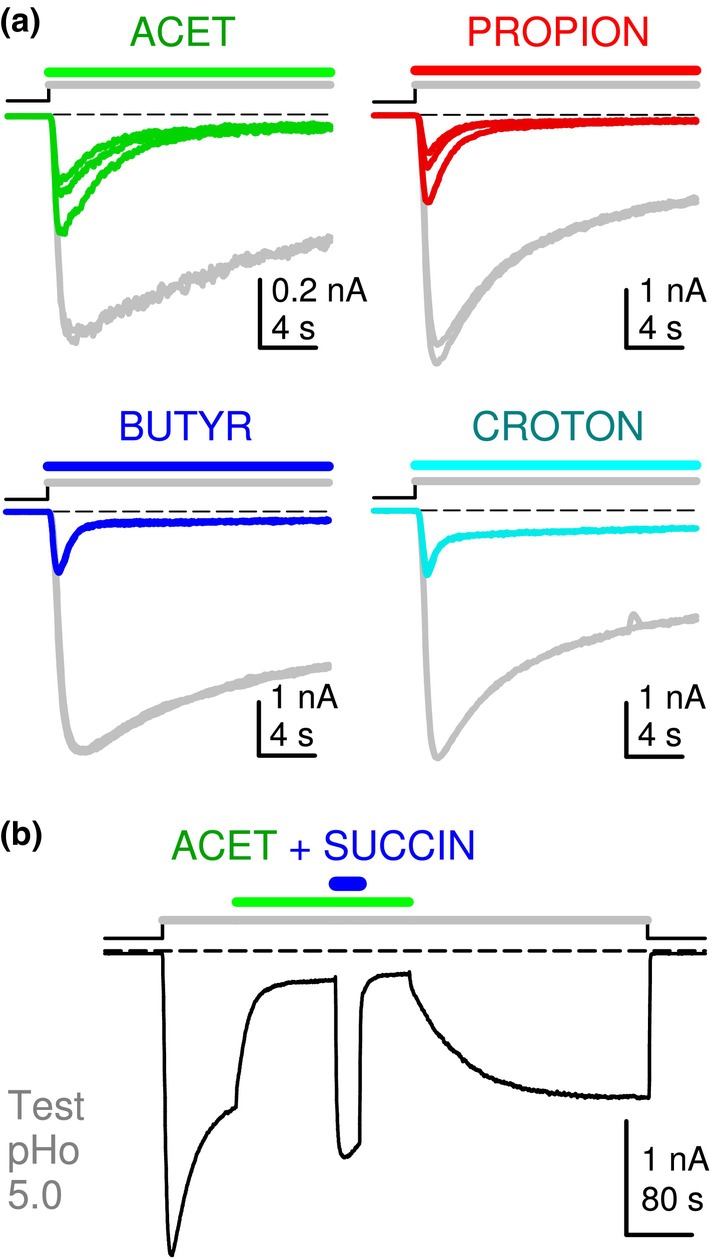
Short‐chain mono‐CBX compounds are negative modulators of allosteric transitions in GLIC. Traces of whole‐cell voltage‐clamp current recorded from tk‐ts13 cells driven to express GLIC, with stimulations from control pHo 7.5. (a) *Sets of superimposed individual current traces* (no averaging) recorded with stimulations at pHo 5.0 (20 s displayed), in the absence and in the presence of acet, propion, butyr, and croton; see compounds in Table 1. Each set is from a different cell. Tests of 30 s duration were applied every 120 s, *that is*, separated by 90 s wash times (no compound, pHo 7.5). Traces in *gray* represent the last two stimulations at pHo 5.0 with no compound, and traces in *color* represent the subsequent first stimulations with a mono‐CBX (1 mM at pHo 5.0; *n* = 3, 3, 2, 2 traces for acet, propion, butyr, and croton, respectively). (b) After GLIC pre‐stimulation at pHo 5.0, acet (1 mM at pHo 5.0) was applied for 120 s, leading GLIC current to decrease to 17% of control. succin (10 mM) was then added to acet (pHo 5.0; 30 s), leading GLIC current to re‐increase to 132% of control (753% of current at 120 s acet). Scale bars, Current: 0.2 nA (acet), or 1 nA (others); Time: 4 s (a), or 80 s (b). Command potential: −40 mV (a) or −30 mV (b).

**FIGURE 2:**
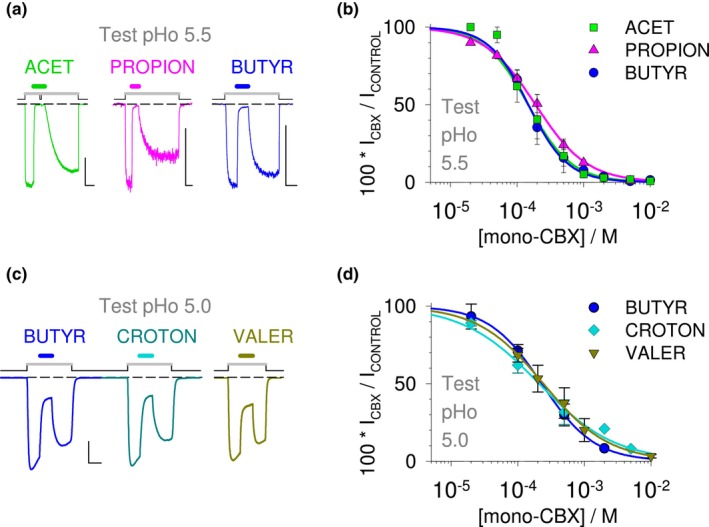
No influence of carbon chain length or double bond on mono‐CBX NAM effect. (a, b) No impact of carbon chain length. (a) Current traces showing activation of GLIC after switching pHo from 7.5 (*Black line* in protocol) to a lower test value (*Gray line* in protocol), here 5.5, followed by a reduction of GLIC current during application of mono‐CBX solutions (*Color lines* in protocol; here at pH 5.5), and recovery from inhibition, followed by deactivation. From left to right are shown effects of the mono‐CBX with two (acet, 5 mM), three (propion, 5 mM), and four carbon (butyr, 2 mM). (b) Corresponding plots of concentration‐to‐steady‐state effect for the three mono‐CBX compounds, tested on GLIC currents at pHo 5.5. Mean value ± SD, constructed from 10 cells (acet), or *n* = 4. (c, d) No impact of the double bond in croton vs. butyr. (c) Current trace from one cell showing equal inhibitory effect relations of 0.5 mM butyr (saturated 4‐carbon mono‐CBX; *Blue line*), and of 0.5 mM croton (with a *trans* double bond; *Cyan line*), on wt GLIC activity at pHo 5.0. The trace with valer (5‐carbon saturated mono‐CBX), also 0.5 mM at pHo 5.0, is from a different cell. (d) Corresponding concentration‐to‐effect plots, showing equal IC_50_s for butyr (*Blue Circle*; *n* = 5) and croton (*Cyan Diamond*; *n* = 4), and for valer (*Dark yellow Triangle*; *n* = 4), at pHo 5.0. Mean value ± SD. Scale bars, Current: 0.1 nA (propion), or 0.5 nA (others); Time: 60 s (acet, propion), or 20 s (others).

**FIGURE 3:**
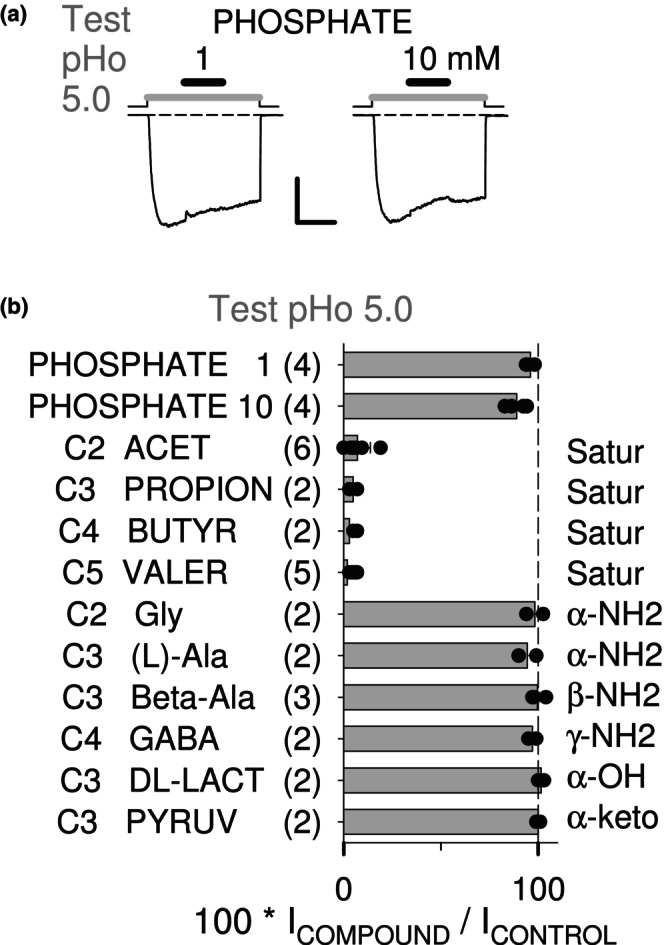
Compound selectivity of the mono‐CBX NAM effect on GLIC. (a) Current traces showing the absence of functional effect on GLIC of phosphate (1 and 10 mM at pHo 5, in 0 Ca solution). Scale bars: 0.4 nA, 20 s. (b) Bar graph of current in the presence of a compound (in % of control pHo 5.0‐elicited current; protocol with pre‐stimulation) for phosphate (1 and 10 mM; in 0 Ca solution), mono‐CBX (1 mM), and mono‐CBX based compounds with amino‐, hydroxy‐, or keto‐ groups (5–10 mM). Each category name also indicates the number of carbons (C2–C5), and the number of cells tested (in brackets). Mean value, with individual cells data points superimposed, and ±SD if *n* = 3 or greater.

**FIGURE 4:**
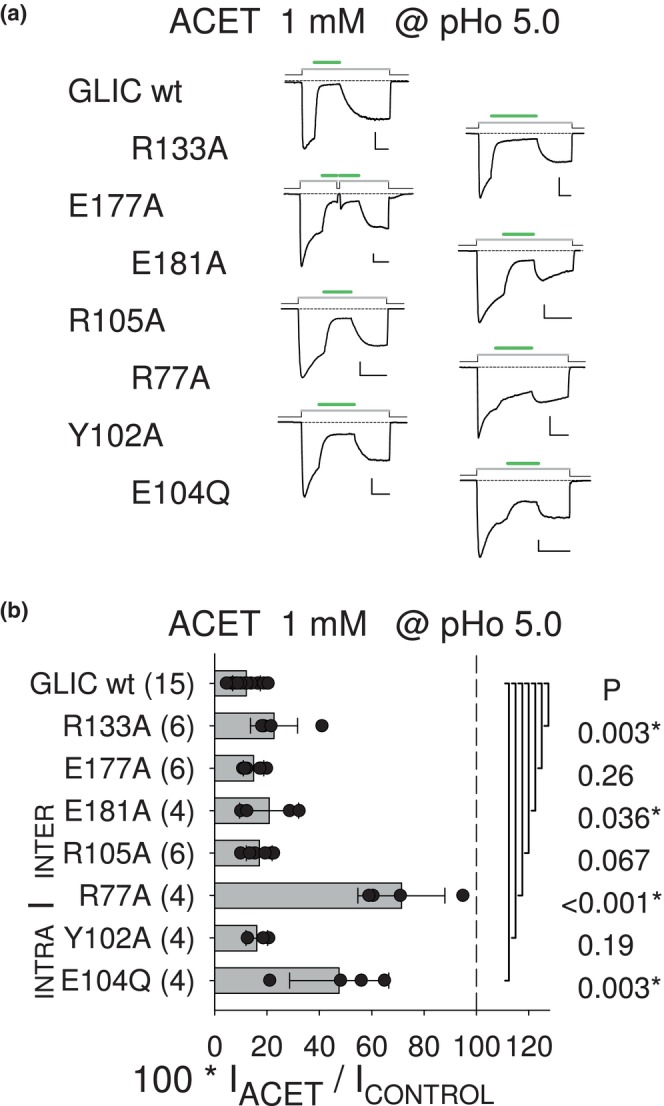
CBX‐binding pockets single mutations: impact on acet NAM effect. (a) Representative current traces illustrating acet tests (1 mM at pHo 5.0) on wt GLIC and single mutation GLIC variants, as indicated left to the traces. Scale bars: 0.2 nA, 60 s. (b) Bar graphs of current measured after 60 s in the presence of 1 mM acet (in % of control at pHo 5.0) on wt and single mutation GLIC variants, as indicated. Residue belonging to the inter‐ or intra‐SU CBX‐binding pocket is indicated, as well as the border/pivot Arg77 (*Bar*). Individual data points are superimposed to bars indicating mean ± SD, for each sample of cells tested, with the number of cells tested given in brackets. Student's *t*‐test *p* value is indicated for each mutant to wt pair of samples.

**FIGURE 5:**
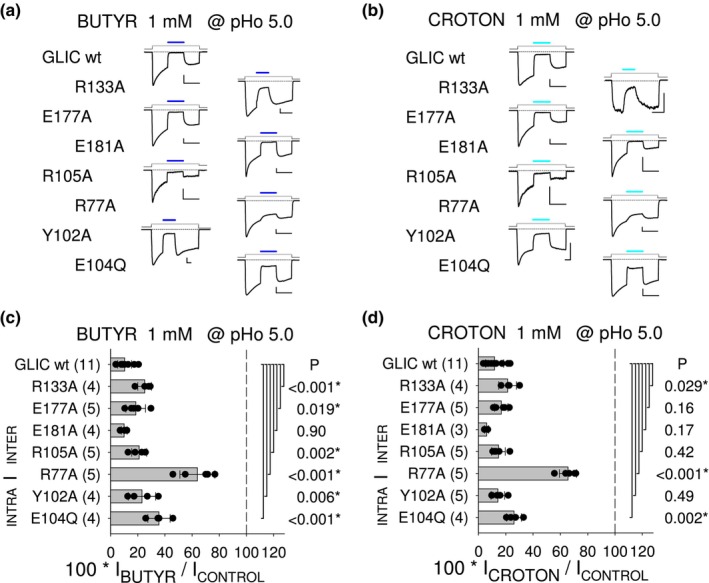
CBX‐binding pockets single mutations: impact on butyr and croton NAM effects. (a, b) Representative current traces illustrating butyr (a) or croton (b) tests (1 mM at pHo 5.0) on wt GLIC and single mutation GLIC variants, as indicated left to the traces. Scale bars, Current: 0.1 nA (butyr R105A; croton R105A and R133A), or 0.4 nA (others); Time: 20 s. (c, d) Bar graphs of current in the presence of 1 mM butyr (c) or croton (d), (in % of control at pHo 5.0), on wt and single mutation GLIC variants, as indicated. Student's *t*‐test *p* value is indicated for each mutant to wt pair of samples.

The intracellular pipette solution was composed of (in mM): CsCl 150, MgCl_2_ 1, HEPES 10, BAPTA 10, CsOH to pH 7.3. The culture dish was washed and filled with the control extracellular solution (in mM): NaCl 165, MgCl_2_ 1, CaCl_2_ 1, MES 10, HEPES 6, NaOH to pH 9.5, then HCl to pH 7.5. Lower pH values were reached by adding to this solution HCl 2 M in water (and more pH 7.5 solution to return pH up if necessary). Various solutions were applied by gravity, near the cell recorded, using either a multiway perfusion system converging to a single tip (50–100 μL/min, exchange time # 1 s) for ancient experiments (Figures 1b, 2, 3, and most of Figures 4 and 5), or a computer‐driven moving‐head multichannel system (RSC‐200; BioLogic, France), exchange time <30 ms in our conditions (Figures 1a, 7, 8 and part of Figures 4 and 5). A 3 M KCl, 5 g/L agar bridge was used to connect the reference electrode to the dish solution. Transmembrane voltage was clamped most of the time to a constant value of −20 mV (occasionally −30 or −40 mV) [except during various non‐detailed controls]. Electric current flowing through the membrane from extracellular to intracellular face (inward current) is counted negative, and represented downwards in the figures.

#### 2.1.4 Two‐electrode voltage‐clamp from oocytes

Whole‐oocyte voltage‐clamp was performed using two 3 M KCl‐filled intracellular pipettes containing Ag/AgCl electrodes, and two extracellular Ag/AgCl pellet electrodes connected to the bath using two separate 3 M KCl‐agar bridges. The control extracellular solution was composed of (in mM): NaCl 100, KCl 3, MgCl_2_ 1, CaCl_2_ 1, MES 10, pH 8.0, in order to keep the conditions used by Nemecz et al. (2017) and Van Renterghem et al. (2023). A different set‐up was used in the present study, with an OC‐725C amplifier (Warner Instruments), and manual fast chamber perfusion with an Omnifit solution exchange system.

### 2.2 Pharmacology and binding sites

As in Van Renterghem et al. (2023), we use the words “ortho*topic*”/”allo*topic*” to characterize the *location* of a binding site, and “allo*steric*” to comment *3D conformational* changes. Therefore (1A), we use “orthotopic site” [in replacement of the more usual “orthosteric site”] to designate the reference agonist site in pLGICs [the neurotransmitter binding site], as well as the homologous location in GLIC [including Arg105, Arg133 (Loop B), Glu177 (Loop C), Glu181]. (1B) According to Sauguet et al. (2013) and Fourati et al. (2020), the inter‐subunit CBX‐binding pocket in GLIC, [including Arg77 (Loop A), Arg105 (Loop E), Glu181 (Loop C), and, for the di‐CBXs, Asn152 (Loop F)], is not exactly coincident with the reference orthotopic site, but situated slightly more deeply, and accessible from the periphery of the pentamer through an entrance which is part of the orthotopic site (Arg133, Glu177). (2) The intra‐subunit CBX‐binding pocket in GLIC [including Arg77 in the apo‐GLIC, and Arg85 (Pre‐β5), Tyr102 (β6), Glu104 (β6)], accessible from the vestibule *lumen*, corresponds exactly to the “vestibular” pocket in ELIC and other pLGICs, and constitutes an “allotopic” binding site, involved in the control of allosteric transitions. When discussing (1) versus (2), we occasionally use “orthotopic” to designate the ensemble (1B + 1A) (inter‐SU CBX‐binding pocket + its orthotopic site entrance), as opposed to the “allotopic,” intra‐SU (= vestibular) pocket (2). In wt GLIC activated at low pHo, keeping as a reference the pLGICs reference binding site, fumarate is an orthotopic PAM, crotonate an orthotopic NAM, propofol an allotopic NAM, extracellular proton an allotopic agonist. The same vocabulary is perfectly consistent regarding Eukaryote pLGICs: neurotransmitters are orthotopic PAMs with agonist property, that is, orthotopic agonists, benzodiazepines are orthotopic PAMs and beta‐carbolines orthotopic NAMs, propofol is an allotopic PAM or NAM, etc. This vocabulary has the advantage of using different words to designate distinct concepts.

In most experiments, a *protocol with pre‐stimulation* was used (Figures 1b, 2, 3, 4, 5, 7, 8). Compound application started after 60 s of GLIC stimulation at low extracellular pH (low pHo) in experiments with acetate 0.5–10 mM (Figures 1b, 2a,b, 4), propionate (Figure 2a,b), and compounds in Figure 3 except for phosphate (20 s) (and after up to 180 s for lower acetate concentrations). For compounds with faster effect (butyrate, crotonate, valerate), pre‐stimulation time was 20 s (Figures 2 and 7 and about two third of cells in Figure 5), and 10 s in Figure 8. Figure 5 includes for each construct about one third of ancient experiments using 60 s pre‐stimulation; then, 20 and 60 s data have been pooled in analysis.

Positive/negative modulation was evaluated as current in the presence of the compound, in percent of the control GLIC current value measured immediately before compound application (with a correction for GLIC current decay only for small inhibitions). The numerical values which are given in text (mean ± SD), used for statistical evaluations, and plotted in graphs are measures of this parameter (100*I_CBX_/*I*_CONTROL_) [occasionally called “PAM *ratio* (%),” and comprised between 100 and 0 in NAM effects]. A *direct protocol* is used in Figure 1a.

Fit of concentration‐effect curves. For proton activation curves, the peak value (*I*pk[pHo]; inward current value at maximal absolute value) within 30 s was considered (*y*), and a plot against H^+^ activity value (*x*) was fitted with a 3‐parameter Hill equation: $y=y_{\max}*\left\{1/\left[1+\left({\mathrm{EC}}_{50}^{n_{H}}/x^{n_{H}}\right)\right]\right\}$, giving *I*_max_, EC_50_, and pH_50_ = −log(EC_50_). For potentiation/inhibition, data for [percent of control current] in the presence of the compound (*y* = 100*I_CBX_/*I*_CONTROL_) to [compound concentration value] (*x*), [which is presented in the figures in log‐scale *x*‐axis display] were fitted using a 4‐parameter Hill equation $\left(\text{potentiation}:y=100+\left(y_{\max}-100\right)*\left\{1/\left[1+\left({\mathrm{EC}}_{50}^{n_{H}}/x^{n_{H}}\right)\right]\right\}\right)$, or a derived sigmoidal function $\left(\text{inhibition}:y=y_{\max}*\left\{1-1/\left[1+\left({\mathrm{IC}}_{50}^{n_{H}}/x^{n_{H}}\right)\right]\right\}\right)$, giving *y*_max_, EC_50_ or IC_50_, and an empirical slope parameter, noted *n*_H_, defined so that its values are positive. For most compounds are presented: a fit of the mean of data (Graphs), and the mean ± standard deviation (SD) of data from individual cells (values in text).

### 2.3 Statistics

In the mutational analysis, the sample of (100*I_CBX_/*I*_CONTROL_) values for each mutant was compared to the sample for wt GLIC (Figures 4 and 5, and part of Figure 7), or the sample for GLIC D86A‐D88A (Figure 8, and part of Figure 7), using Student's *t*‐test. The probability (*p*) for test and reference samples coming from a single normally distributed population are indicated near each mutant data. The *criterium* used in text for a significant impact of a mutation was *p* < 0.05.

## 3 RESULTS

### 3.1 Mono‐CBX compounds NAM effect on wt GLIC

#### 3.1.1 Short‐chain mono‐carboxylates elicit a negative modulation of allosteric transitions in GLIC

A *direct protocol*, where a pHo‐jump (from control pHo 7.5) and compound addition are applied simultaneously, was first used to examine the influence of saturated, short‐chain mono‐CBX compounds on low pHo‐induced GLIC current (Figure 1a).

Using pHo 5.0 (corresponding approximately to pEC_50_ [or pHo_50_] on GLIC), low pHo‐induced GLIC current decay is relatively slow (see control traces, in *gray*, in Figure 1a: more than a half of peak current value remaining after 20s), in comparison with the decay of EC_50_ whole‐cell patch‐clamp currents from most mammalian pLGICs. If a new stimulation includes acetic acid/acetate (acet; 1 mM at pH 5.0), added to the [MES + HEPES] – buffered solution (Figure 1a, *Upper Left* trace), a decreased peak current value, and a faster current decay (than in the absence of acet) are observed, revealing an inhibitory effect of acet.

The 3‐ and 4‐carbon saturated mono‐CBX compounds, propionic acid/propionate (propion), and butyric acid/butyrate (butyr; both 1 mM at pHo 5.0) also elicited a reduced peak current and faster current decay. Alqazzaz et al. (2016) characterized the 4‐carbon mono‐CBX with a *trans* double bond, crotonic acid/crotonate (croton) as a GLIC inhibitor at pH 5.5. croton was also an inhibitor at pHo 5.0 (Figure 1a).

For the first CBX application in each set of Figure 1a, and similar experiments in the direct protocol, the *ratio* of peak current values in the presence and in the absence of compound (Ipk[CBX@5.0]/Ipk[pHo 5.0]) was evaluated. *Ratios* were as follows: 0.400, 0.424, 0.533 (Fig) & 0.575 (acet), 0.380 (Fig) (propion), 0.257 (Fig) & 0.332 (butyr), and 0.228 & 0.244 (Fig) (croton). These small ratios show that a fast inhibition component occurs during GLIC activation in the conditions used. The current (absolute value) time course was fitted with a Hodgkin & Huxley function *m* ^4^ *h* (*m*, a rising exponential function of time; *h*, a decay exponential), giving for current decay (*h*) time constant values of: 2.90, 3.13, 3.33 (Fig) & 5.62 s (acet); 1.47 (Fig) s (propion); 0.541 (Fig) & 0.304 s (butyr); and 0.695 & 0.583 (Fig) s (croton). [*Ratio* and time constant values are ordered according to corresponding cells].

A *protocol with pre‐stimulation* (Bocquet et al., 2007) is used in Figure 1b: GLIC is pre‐stimulated at low pHo (here 5.0) before addition of a compound (here acet 1 mM at pHo 5.0). The 4‐carbon di‐CBX compound succinic acid/succinate (succin) was previously characterized as a positive modulator of the allosteric transitions (PAM) on GLIC activated at pHo 5.0 (Van Renterghem et al., 2023). Applied on a cell after reaching the plateau of inhibition by acet (1 mM at pHo 5.0, 50 s, to 6.6% of control on the cell commented), succin (10 mM at pHo 5.0, without acet), produced a fast, reversible, re‐increase of GLIC current to 143% of the control current recorded immediately before acet (representing 2170% of the current recorded in the presence of acet). A similar pattern was observed if, at the plateau of acet inhibition, succin (10 mM) was co‐applied with acet (1 mM; pH 5.0; Figure 1b): the PAM succin overcame acet inhibitory effect. On three cells, the peak succin current values (co‐application; after 90, 120 & 180 s of acet 1 mM, reaching 5.5%, 17.2% & 11.3% of control current at pHo 5.0) were as follows: 28, 132 (Figure 1b), and 44% of the control current recorded immediately before acet (representing 505, 753 (Figure 1b), and 392% of acet current). Despite variability in its quantitative result, this functional competition experiment shows that inhibition by acet does not occur through an inhibition of permeation, but occurs through a negative modulation of the receptor‐channel gating transitions.

#### 3.1.2 No influence of carbon chain length or double bond on mono‐CBX NAM effect on wt GLIC

A protocol with pre‐stimulation at low pHo was chosen to further characterize mono‐CBX NAM effects on GLIC, as illustrated in Figure 2a for acet (5 mM), propion (5 mM), and butyr (2 mM), at pHo 5.5. The protocol with pre‐stimulation also shows that reversibility occurs after compound wash‐out at low pHo. The relation of acet concentration to minimum inward current value (in percent of control) is plotted in Figure 2b. A sigmoid fit of the mean value (established using 10 cells) against concentration indicates an IC_50_ of 136 μM and a slope parameter equal to 1.6. Fitting the concentration to current (% value) relations from individual cells indicated an IC_50_ of 128 μM (±55 μM, *n* = 5) for acet at pHo 5.5. With 3‐ and 4‐carbon compounds, the IC_50_ and slope parameter values obtained from individual cells were for propion 204 μM (±51 μM, *n* = 4) and 1.1 (±0.1, *n* = 4), and for butyr 150 μM (±23 μM, *n* = 4) and 1.5 (±0.2, *n* = 4).

Changing the test pHo value from 5.5 to 5.0 had a significant but weak influence on butyr effect (Figure 2c,d), with an IC_50_ value of 219 μM (±31 μM, *n* = 4; *p* = 0.012) at pHo 5.0 versus pHo 5.5. The 5‐carbon mono‐CBX compound, valeric acid/valerate (valer) showed an inhibitory effect, with an IC_50_ of 289 μM (±116 μM, *n* = 4). croton NAM effect at pHo 5.0 (Figure 2c,d) was indistinguishable from butyr effect at the same pHo, with an IC_50_ value of 191 μM (±63 μM, *n* = 4; *p* = 0.46). Isocrotonic‐acid/isocrotonate, the 4‐carbon mono‐CBX with a double bond in the *cis* configuration, is known to be unstable due to spontaneous *cis* to *trans* isomerization at low pH. Having no simple tool to evaluate the proportion of *cis* compound, we decided not to test isocrotonate.

These data show that mono‐CBX compounds negatively modulate GLIC with no impact of the carbon chain length, no impact of a *trans* double bond in the 4‐carbon compound, and no major influence of the test pHo value between 5.5 and 5.0. Using a pipette solution at pH 7.3, we found no condition in which acet, propion, butyr, or croton would produce a potentiation of wt GLIC current, no evidence for a biphasic effect according to concentration or pHo: only inhibitory effects were observed with mono‐CBX compounds on wt GLIC.

#### 3.1.3 Selectivity of GLIC for mono‐carboxylates

A protein crystallization liquor buffered with phosphate, instead of acetate or another CBX, was used by Fourati et al. (2015) to obtain an Apo‐GLIC crystal structure (PDB reference 4qh5), and no phosphate ions were identified in the structure. As an echo, we performed functional tests of phosphate on GLIC activity (recorded in a solution identical to our extracellular solution except that CaCl_2_ was omitted): as shown with current traces (Figure 3a), phosphate (1 and 10 mM) had no effect on GLIC activity at pH 5.0 (*n* = 4 cells with two concentrations each; Figure 3b).

Tested at 1 mM with pre‐stimulation, acet and propion were active at pHo 5.0 (Figure 3b), as seen for butyr and valer (Figures 2c,d and 3b). Neighbor compounds as well (5 or 10 mM) were tested with pre‐stimulation at pHo 5.0 (Figure 3): the amino‐derivatives glycine (C2 as acet) and (l)‐alanine (C3 as propion), β‐alanine (β‐amino propionate) and γ‐amino butyrate (GABA), as well as the α‐hydroxy‐derivative DL‐lactate (C3 as propion) and α‐keto‐derivative pyruvate (C3 as propion). All these compounds had no effect on GLIC current (2–5 cells each), showing a strong chemical selectivity for the short‐chain mono‐CBXs NAM effect on GLIC.

#### 3.1.4 Functional relevance of GLIC CBX‐binding pockets for negative modulation by acet, butyr, and croton: Mutational analysis (pHo 5.0)

The inter‐SU (orthotopic) and intra‐SU (vestibular) CBX‐binding pockets revealed in the GLIC protein by crystallographic structures (Fourati et al., 2020; Sauguet et al., 2013), and the orthotopic pocket entrance, were previously evaluated (at pHo 5.0) for their involvement in the PAM effects of succin and fumaric acid/fumarate (fumar), and the NAM effect of caffeic acid/caffeate (caffe). Van Renterghem et al. (2023) showed that a mutation in anyone of these locations has an “all‐or‐none” impact, with suppression of both the 4‐carbon di‐CBX PAM effects, and (except for E181A) the caffe NAM effect. Here, we used the same single mutants to test (also at pHo 5.0) the functional relevance of these binding *loci* in the 4‐carbon mono‐cbx NAM effects and acet effect. We show that most mutations have much less impact on the mono‐CBX NAM effects, than on di‐CBX and caffe effects.

acet (Figure 4) and the 4‐carbon compounds butyr and croton (Figure 5) were tested on the CBX‐pockets single mutants, at 1 mM with pre‐stimulation at pHo 5.0. The mutational analysis results illustrated with current traces (Figures 4a and 5a,b), and displayed as bar graphs (Figures 4b and 5c,d), are as follows. (1) The greatest impact on their ability to inhibit GLIC occurred with the removal of the pivot residue Arg77. The data on R77A were as follows: in the presence of acet 71.4% of control (±16.6%, *n* = 4; *p* < 0.001), with butyr 63.9% (±12.9%, *n* = 5; *p* < 0.001), and with croton 65.6% (±6.3%, *n* = 5; *p* < 0.001), versus 12.2% of control (±5.3%, *n* = 15), 10.3% (±5.1%, *n* = 11), and 11.9% (±6.6%, *n* = 11), respectively, on wt GLIC. (2) The intra‐SU CBX‐binding pocket residue Glu104 was next, as acet NAM effect was strongly reduced on E104Q, with, in the presence of acet 47.6% of the control current (±18.9%, *n* = 4; *p* < 0.001). E104Q had a significant impact on butyr (35.6% of control, ±8.2%, *n* = 4; *p* < 0.001) and croton (26.1%, ±5.3%, *n* = 4; *p* = 0.002) NAM effects. (3) The other intra‐SU pocket mutation, Y102A, had little or no impact on acet (16.1%, ±4.2%, *n* = 4; *p* = 0.19), butyr (23.1%, ±10.1%, *n* = 4; *p* = 0.006), and croton (14.2%, ±5.0%, *n* = 5; *p* = 0.49) effects. (4) Unexpectedly, the inter‐SU CBX‐binding pocket mutation R105A had little or no impact on the mono‐CBX NAM effects, with the following data: with acet 17.1% of control (±4.9%, *n* = 6; *p* = 0.067), with butyr 21.0% (±5.6%, *n* = 5; *p* = 0.002), and with croton 14.7% (±5.0%, *n* = 5; *p* = 0.42).

(5) The E181A substitution, known to have on GLIC no impact on caffe NAM effect (Prevost et al., 2013, pHo 5.5; Van Renterghem et al., 2023, pHo 5.0), and no impact on croton NAM effect (Alqazzaz et al., 2016, pHo 5.5), had no impact either on the NAM effect of acet (Figure 4), butyr, and croton (Figure 5) in our wt GLIC data at pHo 5.0. These data (no impact on NAM effects) are related to the fact that, with the 4‐carbon di‐CBX, E181A not only abolished the PAM effect but also revealed a unique di‐CBX NAM effect (see figure 7 in Van Renterghem et al., 2023). (6) Regarding the orthotopic site entrance mutations, R133A had a minor significant impact in this protocol (see Section 4): with acet, 22.7% (±9.0%, *n* = 6; *p* = 0.003); with butyr, 25.1% (±5.1%, *n* = 4; *p* < 0.001); with croton, 21.4% (±6.6%, *n* = 4; *p* = 0.029), as compared to the respective wt values, 12.2% of control (±5.3%, *n* = 15), 10.3% (±5.1%, *n* = 11), and 11.9% (±6.6%, *n* = 11), already given above. E177A had almost no impact: with acet, 14.9% of control (±3.9%, *n* = 6; *p* = 0.26); with butyr, 18.6% (±7.2%, *n* = 5; *p* = 0.019); and with croton, 16.8% (±4.8%, *n* = 5; *p* = 0.16).

A “loose” pattern of mutational impact is therefore observed for the mono‐CBX NAM effects, characterized by a weak impact (except for R77A) of every single mutation in the inter‐SU (orthotopic) pocket or its orthotopic entrance, or in the intra‐SU (vestibular) CBX‐binding pocket.

### 3.2 A double mutation in Loop Ω favors positive modulation

#### 3.2.1 The D86A‐D88A pre‐β5 double mutation has a week loss of function impact on proton‐elicited activation

**FIGURE 6:**
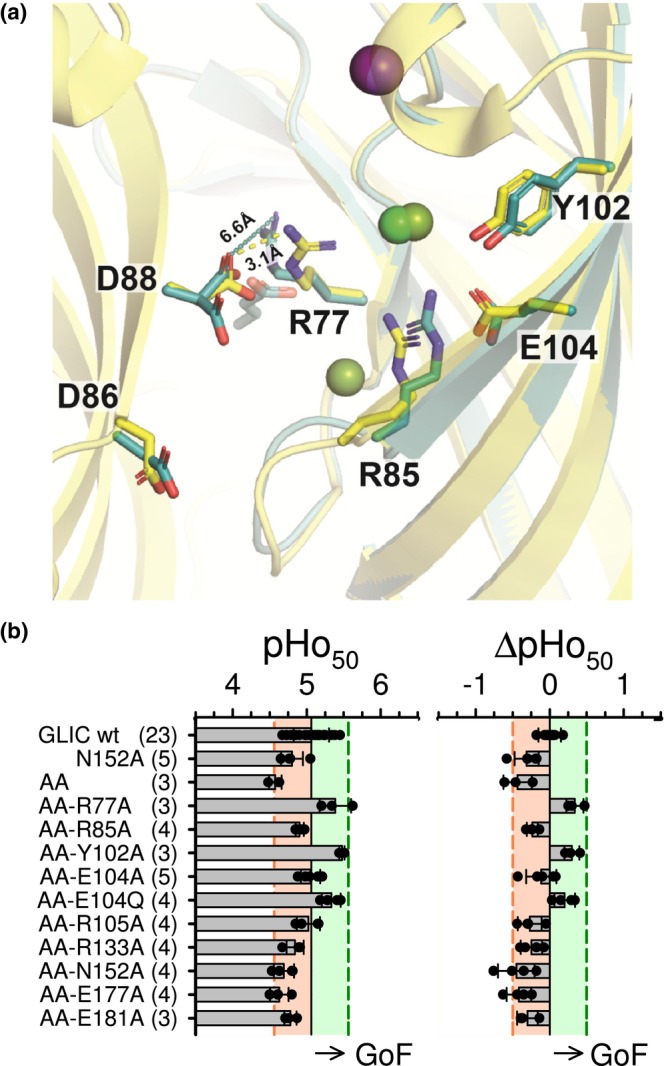
The pre‐β5 strand double mutation D86A‐D88A (AA): location, and low impact on GLIC proton sensitivity. (a) Location of Asp86 and Asp88 in GLIC crystal structure. Superimposed views from the Apo‐GLIC (crystals grown in Phosphate buffer) structure (4qh5; *Yellow*), and the GLIC‐croton co‐crystal structure (6hji; *Dark* c*yan*). PDB references from Fourati et al. (2015, 2020), respectively. Views are in GLIC ECD, from the axis of the pentamer toward periphery, with pre‐β5 strand (*n*) on the left (bearing Asp86 and Asp88), and intra‐SU pocket (*n* + 1, *clockwise*) on the right. A chloride ion occupies the intra‐SU pocket (“CBX‐empty”) in the apo‐GLIC structure (*Green sphere*) and the croton structure (*Yellow green sphere*). A second chloride (left to Arg85) is present in the croton structure (*Yellow green*). Whereas, in 6hji only, the inter‐SU pocket (not represented) is occupied by a croton molecule, represented behind the Asp88 carboxyl group. A sodium ion is represented (*Light Purple sphere* in the Apo‐structure, *Dark purple sphere* in croton structure). An Asp88(*n*)‐Arg77(*n* + 1) ion bridge (3.1 Å), present in the Apo‐structure, is released in the croton structure (6.6 Å). (b) Two‐electrode voltage‐clamp data obtained using the *Xenopus* oocyte expression system. Bar graphs showing values of the pHo corresponding to half maximal activation by low‐pH extracellular solutions (pHo_50_, *Left*), and the difference between pHo_50_ values from mutant and wt GLIC (ΔpHo_50_, *Right*); wt reference: recording on the same day or the day before, from one or two oocytes of the same batch, and in the same solutions as for the mutant considered. For each mutant is shown the data from 3 to 5 oocytes, coming from at least two injections. Pink and green colored areas indicate the mutant inclusion criteria: ±0.5 pH unit from the wt pHo_50_ value (EC_50_ *ratio* 0.3–3). In this representation, the data for a gain of function (GoF) variant go right to wt data.

The pre‐β5 strand, lining the extracellular vestibule lumen, is also part of the wall of the intra‐SU CBX‐binding pocket on the axial side of GLIC ECD [opposite to Arg77 and the inter‐SU CBX‐binding pocket] (Fourati et al., 2015, 2020; Sauguet et al., 2013; see Figure 6a and Figures S1 and S2). Indeed, the pre‐β5 strand includes Arg85, whose side chain belongs to the intra‐SU pocket; Arg85 side‐chain coordinates the intra‐SU bound CBX if any and is otherwise hold in place by Glu104 and a chloride (Fourati et al., 2020). Adjacent within the pre‐β5 strand is the pair of aspartate residues, Asp86 and Asp88, pointing opposite, toward the vestibule lumen, where they bind cations in GLIC crystal structures (Fourati et al., 2015; Sauguet et al., 2013). The pre‐β5 strand appears located somehow in‐between the CBX‐binding pockets and the gating machinery, as the pre‐β5‐β5‐β6 “sandwich” ends down at the ECD–TMD interface with the β6‐β7 Loop, or Pro‐Loop (Jaiteh et al., 2016), essential to gating. [In Eukaryote pLGICs, the Pro‐Loop, stabilized by a disulfide bridge, is usually called Cys‐Loop]. The pre‐β5 strand in GLIC corresponds to the pLGICs Loop Ω defined by Hu et al. (2018), and analyzed systematically in Eukaryote pLGICs structures by Brams et al. (2020).

Regarding GLIC activation by protons (characterized using the *Xenopus* oocyte expression system), the pre‐β5 strand double mutation D86A‐D88A (AA) produced a slightly loss of function (LoF) GLIC variant (Figure 6b), with ΔpHo_50_ = −0.43 (±0.19, *n* = 3 [2 inj]), [in comparison with data recorded from oocytes expressing wt GLIC on the same day or the day before]. These data are consistent with the data published by Nemecz et al. (2017). To ensure that the impact of D86A‐D88A on GLIC modulation by the mono‐CBX was not a simple consequence of this LoF property, we included here another LoF mutant, N152A (ΔpHo_50_ = −0.31 ± 0.16, *n* = 5 [2 inj]; Figure 6b). Asn152 has a special interest, as it belongs to the inter‐SU pocket, but has no contact with the inter‐SU bound CBX when it is a mono‐CBX molecule: Asn152 coordinates the second carboxyl group of the di‐CBX molecule (Fourati et al., 2020). Consistently, in addition to the mono‐CBXs, we tested here the di‐CBXs and caffe.

#### 3.2.2 The D86A‐D88A pre‐β5 double mutation favors compound‐elicited positive modulation

**FIGURE 7:**
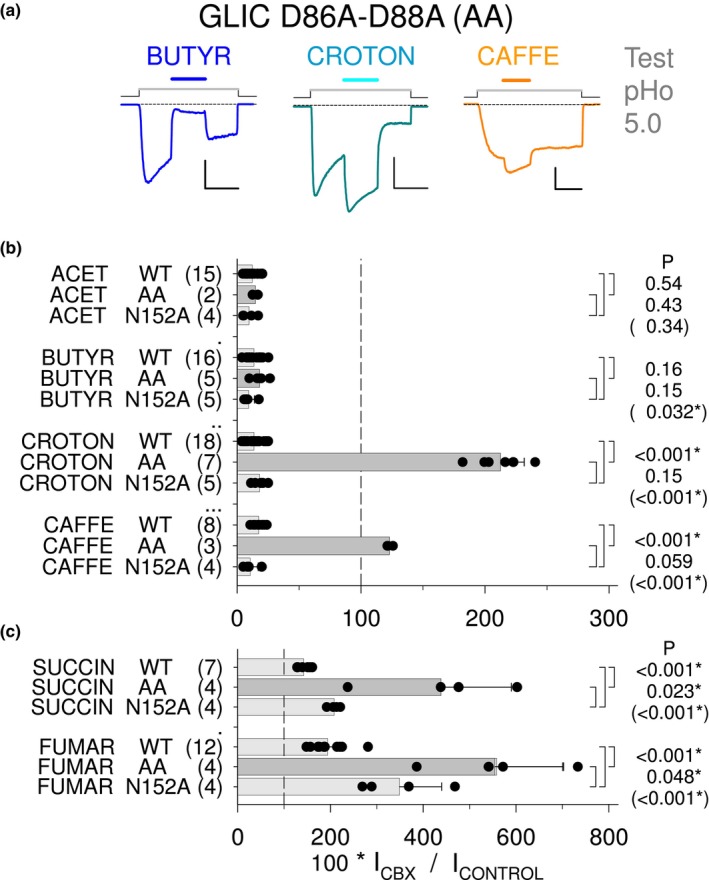
The pre‐β5 strand double mutation D86A‐D88A (AA): major impact on compound‐elicited modulation, and comparison with N152A. (a) Current traces from three cells driven to express GLIC D86A‐D88A (GLIC AA), showing the unchanged NAM effect of butyr (1 mM), the inverted, strong PAM effect of croton (1 mM), and inverted, weak PAM effect of caffe (0.1 mM), all at pHo 5.0. See Figure 2c or Figure 5a,b for current traces showing the NAM effects of butyr and croton on wt GLIC at pHo 5.0. On GLIC AA, croton behaved as a PAM, but had no agonist property (see Text, Section 3.2.2). Scale bars: 0.4 nA, 20 s. (b, c) Bar graphs showing the current values (in % of control at pHo 5.0) obtained from cells expressing wt GLIC, or D86A‐D88A, or N152A, in the presence of (b) a mono‐CBX (acet, butyr, croton; 1 mM), or caffe (0.1 mM), or (c) a di‐CBX compound (succin, fumar; 10 mM), all at pHo 5.0. On the pre‐β5 variant AA, effects of croton and caffe are inverted, from near‐full negative modulation on the wt, to positive modulation on AA, whereas butyr and acet NAM effects are unchanged. succin and fumar PAM effects are increased on the pre‐β5 double mutant AA. As is GLIC AA, N152A is a slightly loss‐of‐function variant (Figure 6b). However, the N152A mutation has no impact on croton and caffe NAM effects (b), whereas N152A increases the di‐CBX PAM effect, but much less than AA (c). Among variants with a slightly loss‐of‐function property regarding GLIC activation by protons, N152A was chosen because, in the GLIC‐CBX co‐crystal structures (Fourati et al., 2020), Asn152 side chain is in contact with the second carboxyl group of the di‐CBX compounds, but has no contact with the mono‐CBX compounds. Statistics: *t*‐test *p* values are indicated (top‐down) for AA to wt, N152A to wt, and N152A to AA (in brackets) sample comparisons.

Compound modulation of the D86A‐D88A pre‐β5 variant (AA), N152A mutant and wt GLIC, was investigated in the protocol with pre‐stimulation at pHo 5.0 (Figure 7). Surprisingly, croton effect (1 mM) was inverted on the pre‐β5 double mutant, from an almost full inhibition at 13.6% of control (±6.1%, *n* = 18) on the wt, into a strong potentiation at 232% of control (±59%, *n* = 8; *p* < 0.001) on the D86A‐D88A mutant (Figure 7a,b). Such an inversion did not occur with butyr, the corresponding but saturated 4‐carbon mono‐CBX compound (Figure 7a,b). acet and butyr remained inhibitors on AA, with no significant change for acet (on wt: 12.2%, ±5.3%, *n* = 15; on AA: 14.6%, ±3.0%, *n* = 2; *p* = 0.54) and butyr (on wt: 13.6%, ±5.9%, *n* = 16; on AA: 18.0%, ±6.0%, *n* = 5; *p* = 0.16). caffe effect, however, was also inverted, from an almost full inhibition at 17.4% of control (±4.6%, *n* = 8) on the wt, into a mild potentiation at 123% (±3%, *n* = 3; *p* < 0.001) on AA (Figure 7b). croton (1 mM) was inactive at pHo values 7.5, 7.0, and 6.0 (*n* = 2 cells each); it is therefore not an agonist on the AA variant. The other LoF mutation, N152A, had no significant impact on the negative modulation by croton and the three other NAMs: for acet 9.7% of control (±5.8%, *n* = 4; *p* = 0.43), for butyr 9.3% (±4.8%, *n* = 5; *p* = 0.15), for croton 18.1% (±5.4%, *n* = 5; *p* = 0.15), and for caffe 10.6% (±6.5%, *n* = 4; *p* = 0.059) (Figure 7b). The inverted PAM effect of croton and caffe is therefore not determined by the weak LoF property, but specific to the pre‐β5 double mutation.

The PAM effects of succin and fumar were strongly increased in the pre‐β5 AA variant (Figure 7c): for succin, 438% of control (±151%, *n* = 4; *p* < 0.001) on AA, versus 142% (±14%, *n* = 7) on the wt; for fumar, 558% of control (±142%, *n* = 4; *p* < 0.001) on AA, versus 194% (±36%, *n* = 12) % on the wt (Figure 7c). Unexpectedly, succin and fumar PAM effects were also *increased* on the N152A mutant (but to a lesser extent than in AA): on N152A, with succin, 208% (±12%, *n* = 4; *p*_152vswt_ < 0.001, *p*_152vsAA_ = 0.023); with fumar, 349% (±91%, *n* = 4; *p*_152vswt_ < 0.001, *p*_152vsAA_ = 0.048) (Figure 7c).

#### 3.2.3 Functional relevance of GLIC cbx‐binding pockets for croton PAM effect on the pre‐β5 GLIC variant D86A‐D88A: Mutational analysis on an AA basis (pHo 5.0)

The residue dependency analysis (Figures 4 and 5) showed that single mutations in the CBX‐binding pockets have a weak impact (except for R77A) [“loose” pattern] on the mono‐CBX NAM effects, contrasting with their “all‐or‐none” impact on the di‐CBX PAM effects (see figure 7 in Van Renterghem et al., 2023). As croton is converted into a PAM on the AA pre‐β5 variant (Figure 7a,b), we decided to check the impact of adding single mutations in the CBX‐binding pockets (on an AA basis). Would croton PAM effect on AA display a mono‐CBX/NAM type “loose” pattern (according to compound structure)? Or would it display a di‐CBX/PAM type “all‐or‐none” pattern of impact (according to compound effect)? We therefore added the pre‐β5 double mutation to every CBX‐binding pocket single mutant.

**FIGURE 8:**
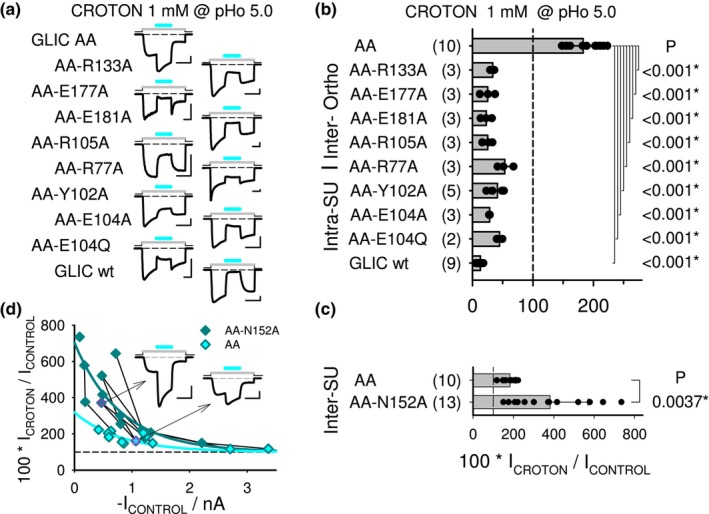
CBX‐pockets single mutations added to the AA variant: impact on croton inverted, PAM effect. (a) Representative current traces illustrating croton tests (1 mM at pHo 5.0) on GLIC AA, triple mutation GLIC variants, and wt GLIC, as indicated left to the traces. Scale bars, Current: 0.2 nA (AA‐E177A), 1 nA (AA‐R77A), or 0.4 nA (others); Time: 10 s. (b) Bar graph of current in the presence of croton (1 mM; in % of control at pHo 5.0), on the pre‐β5 double mutant D86A‐D88A (AA), on the triple mutation GLIC variants, and on wt GLIC. Regarding the CBX‐binding pocket single mutations, croton PAM effect on the pre‐β5 double mutant shows the “all‐or‐none” pattern of residue dependency, previously observed with the di‐CBX PAM effect (see figure 7 in Van Renterghem et al., 2023): any croton PAM effect is lost with anyone of the single CBX‐binding sites mutations. However, with the CBX‐pockets mutations, a croton NAM effect reappears, showing a “loose” pattern of residue‐dependency, as with acet, butyr, and croton NAM effect on wt GLIC (see Figures 4 and 5). Statistics: each triple mutant sample of data was compared to the AA double mutant sample. (c) Bar graph of current in the presence of croton (1 mM; in % of control at pHo 5.0) on the pre‐β5 double mutant D86A‐D88A without (AA) and with the N152A mutation (AA‐N152A). (d) The croton data shown in *C* were plotted against the control current amplitude (absolute value) for AA‐N152A (*Dark Green* diamond) and AA (*Cyan* diamond). Data points obtained on the same day of recording are joined by a black line. The current traces displayed as *Inset* were recorded, on the same day, from an AA‐N152A cell (*Left* trace) and an AA cell (corresponding data points spotted in pink in plot). The PAM ratio (in %) decreases with increasing current amplitude. A single exponential decay function (*Dark Green* line) (decaying toward *y*_0_ = 100%, fixed) was fitted to the AA‐N152A data, giving a decay constant tau = 800 nA, and an amplitude (at 0 nA) of 604%. The AA data, arbitrarily fitted (*Cyan* line) with the same decay constant value (800 nA, fixed) gave an amplitude (at 0 nA) of 219%. The data in (c), and its further analysis in (d), clearly show that the N152A mutation unexpectedly increases croton PAM effect on the AA variant (as N152A unexpectedly increases the di‐CBX PAM effects on wt GLIC, Figure 7c). On GLIC AA‐N152A, croton behaved as a PAM, but had still no agonist property (see Text).

The triple mutants were first characterized for their sensitivity to pHo (Figure 6b). Expressed in *Xenopus* oocytes, they were all functional, and presented pHo_50_ values within the mutant inclusion *criterium*, that is, within ±0.5 pH unit from the pHo_50_ value measured for wt GLIC [with the same batch of oocytes and solutions]. Therefore, the triple mutants were all included in the study.

croton was tested in whole‐cell patch‐clamp, in the conditions previously used for the CBX‐pocket single mutants. croton effect on each [AA + CBX‐pocket] triple mutant was compared to its effect on the AA double mutant (Figure 8). Adding anyone of the binding pocket mutations (excluding AA‐N152A) fully abolished croton PAM effect, despite the presence of the pre‐β5 double mutation (Figure 8a,b). Therefore, croton PAM effect (inverted on AA, vs. wt) clearly displays a di‐CBX/PAM type pattern, with an “all‐or‐none” impact of every binding pocket mutation [according to a CBX PAM effect]. Here however, with AA, croton ability to positively modulate GLIC being fully abolished (by any binding pocket mutation), its ability to negatively modulate GLIC is then revealed. In addition, the recovered negative modulation by croton displays the mono‐CBX/NAM type “loose” pattern of impact [as now compared with wt GLIC] (Figure 8b), similar to the pattern observed when adding the single mutations to GLIC wt (see Figures 4 and 5): Each mutation had a weak impact; greatest impact observed with R77A (removing the pivot); next greatest impact with an intra‐SU pocket mutation; weak significant impact of orthotopic site mutations R133A or E177A, and inter‐SU pocket mutation R105A; no impact of E181A [*i*.*e*., according to a mono‐CBX structure].

Finally, the N152A mutation, which had no impact on croton NAM effect (wt basis; Figure 7b) but increased succin and fumar PAM effects (wt basis; Figure 7c), also increased croton PAM effect (AA basis; Figure 8c). The potentiating impact of N152A on an AA basis was significant (381% ± 189%, *n* = 13; *p*_AA152vsAA_ = 0.0037, vs. 183% ± 28%, *n* = 10 for AA, as mentioned). However, as the values for croton effect on AA‐N152A were noticeably dispersed, we decided to plot the PAM ratio in % (=100*I_CROTON_/*I*_CONTROL_) according to the control current amplitude (absolute value). This representation shows that the PAM ratio (%) decreases with increasing control amplitude. It also makes clear that the PAM ratio (%) value is consistently increased in AA‐N152A versus AA. croton is still not an agonist on the AA‐N152A variant, as it remained inactive at pHo values 7.5, 7.0, and 6.0 (1 mM; *n* = 2 cells each).

## 4 DISCUSSION

### 4.1 Short‐chain mono‐CBXs as negative modulators of allosteric transitions in GLIC

We report that short‐chain, saturated mono‐CBX compounds are negative modulators of the allosteric transitions in GLIC. The NAM effect of acet, propion, butyr, or valer (2‐ to 5‐carbon compounds) occurs with no influence of the carbon chain length on the concentration—effect curve (Figure 2). croton was previously published to negatively modulate GLIC (Alqazzaz et al., 2016). Our data show that the double bond present in croton, not in butyr, has no influence on the concentration—NAM effect curve (Figure 2c,d). These properties contrast with the fact that a 4‐carbon di‐CBX is a better PAM than a 3‐ or 5‐carbon di‐CBX, and fumar (with double bond) a better PAM than succin (see figures 1A,B and 2A–D in Van Renterghem et al., 2023). In addition, both data are consistent with the previously published GLIC‐CBX co‐crystal structures (Fourati et al., 2015, 2020; Sauguet et al., 2013), showing that the mono‐CBXs are bound by a single end of the molecule (the carboxyl group), whereas the di‐CBX compounds are bound by both ends.

Mutations located away from the pore or vestibule lumen (R77A, E104Q) abolish the major part of the mono‐CBX inhibitory effect (Figures 4 and 5), suggesting that inhibition is not due to a channel block or another permeation mechanism. The fact that succin PAM effect overcomes acet inhibitory effect (Figure 1b) also excludes a permeation mechanism. In addition, the D86A‐D88A double mutation inverts inhibition to a potentiation (croton and caffe, Figure 7). The data therefore demonstrate that, at least for croton and caffe, we are actually dealing with a compound‐binding elicited negative modulation of GLIC gating.

### 4.2 Inhibition by CBX compounds and GLIC current decay

Given their low p*K*a values, carboxylic acids have been reasonably chosen as low‐pH buffers in some functional studies of GLIC. The mono‐CBX NAM property then produces a (relatively) fast GLIC current decay (due to compound inhibition, as in Figure 1a), which may be misinterpreted as low pHo related GLIC desensitization. CBX compounds cannot be used as pH buffers in functional studies of GLIC, and previously published reports using acet as buffer need to be reinterpreted. The tri‐CBX citrate, used by some authors, had no effect on GLIC current, but produced some instability in the recording. We chose to keep‐on with Good's buffers (thought to be non‐membrane‐permeant), despite the fact that the lowest p*K*a value available (near 6.2 for MES) gives a poor buffering capacity at the low pHo values used with GLIC. This point is counter‐balanced by the use of continuous extracellular solution flow in electrophysiological recordings.

Regarding GLIC desensitization, our data show that, in the absence of a NAM compound, low pHo‐induced GLIC current decay is very slow (Figure 1a Currents in *gray*). As mentioned by other authors, it is also very variable. Van Renterghem et al. (2023, Figure 3 and text) proposed that a progressive (and variable), low pHo‐induced, *drop in intracellular pH* may be the major determinant of GLIC current decay kinetics (at least in the absence of compound). According to the preliminary data published by Hilf and Dutzler (2009), with pHi = 4, GLIC is no more activated by its “agonist,” a pHo 4 extracellular solution (the point, however, requires further investigation). Would GLIC at low pHi correspond to its desensitized state? Whether a desensitized state exists or not in GLIC becomes a question of definition. And whether a desensitized state (defined as in Eukaryote pLGICs) may be favored by orthotopic binding of a PAM compound, such as fumar, remains an unanswered question.

### 4.3 A double bond favors positive modulation of allosteric transitions, and is associated with exclusive inter‐SU binding in the structures

Regarding the 4‐carbon compounds, the double bond favors the PAM effect of a di‐CBX (fumar vs. succin; see figures 1A,B and 2C,D in Van Renterghem et al., 2023), not the NAM effect of a mono‐CBX (butyr and croton have indistinguishable NAM effects on GLIC wt; Figures 2c,d, 5, 7b). GLIC binding sites, however, are able to distinguish croton from butyr, at least in the pre‐β5 variant, since croton modulatory effect is then inverted, whereas butyr NAM effect is unchanged (Figure 7a,b). Here, a double bond is required for the 4‐carbon mono‐CBX to be converted into a PAM on the pre‐β5 variant. As is the case for croton and fumar, the caffe molecule has a *trans* double bond in *alpha* to its carboxyl group. Consistent with our conclusion, caffe effect was also inverted to a PAM on the pre‐β5 variant (Figure 7a,b). From these functional data, we conclude that a double bond has no impact on NAM effects, but favors PAM effects.

In the crystal structures of GLIC‐CBX complexes (Fourati et al., 2020; Sauguet et al., 2013; see Table 1), the 2‐ or 3‐carbon mono‐CBX compounds (acet, propion) and the saturated 4‐carbon di‐CBX (succin) are found occupying the two CBX‐binding pockets. In contrast, the 4‐carbon compounds with a double bond (croton, fumar), both occupying the inter‐SU pocket, are absent from the intra‐SU pocket (see Table 1 & Figure 6a), suggesting that the vestibular pocket cannot handle the compounds with a double bond. No co‐crystal structure is available for butyr or caffe; but from the available structures, it may be expected that the saturated 4‐carbon mono‐CBX would probably be found in the two pockets (as acet, propion, and succin), but caffe (with its C2–C3 double bond) only in the inter‐SU pocket.

### 4.4 The “all‐or‐none” pattern of mutational impact for croton inverted effect supports the model with inter‐SU binding and vestibular control

**FIGURE 9:**
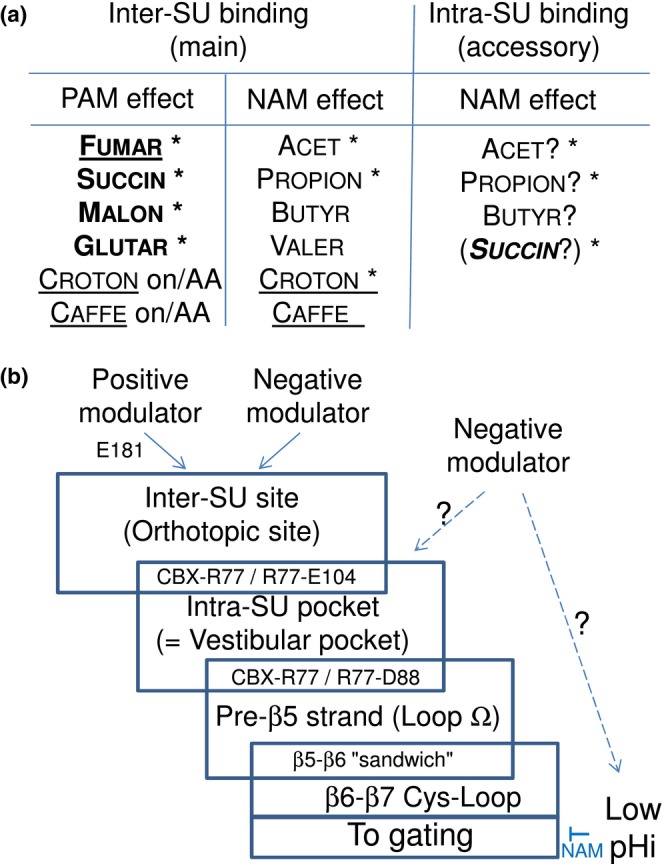
A mechanistic hypothesis compatible with all crystallographic and electrophysiological CBX data on GLIC. A mechanistic hypothesis compatible with all CBX data on GLIC (b), and suggested ligands to the two CBX‐binding pockets (a). In (a), we summarize the respective involvement of various compounds, suggested by crystallographic data (Fourati et al., 2020; Sauguet et al., 2013; see also Table 1) and electrophysiology data (Van Renterghem et al., 2023, and the present report). Codes in A: *Star, presence observed in a co‐crystal structure; *Bold*, a di‐CBX; *Underlined*, with a *trans* double bond (thought to bind only to inter‐SU site); AA, effect on the pre‐β5 double mutant AA; *In brackets*, hypothetic secondary NAM influence of a di‐CBX, limiting its PAM effect;? *Question mark*, compatible with, not demonstrated by functional data. In (b), Starting with the single occupancy model, with two sites “in series,” and vestibular control of the coupling between (the main) orthotopic/inter‐SU ligand binding and gating, proposed by Van Renterghem et al. (2023), we add here (1) an involvement of the pre‐β5 strand (Loop Ω), (2) an accessory intra‐SU site binding putatively contributing to negative modulation, and (3) a putative contribution of mono‐CBX‐induced sub‐membrane pH lowering. From our data, we suggest that only inter‐SU binding may lead to a PAM effect.

The spectacular inversion of croton effect (not butyr effect) on the D86A‐D88A pre‐β5 variant (Figure 7a,b), together with the exclusive inter‐SU binding of croton in the available structure (see Figure 6a), support the involvement of an (exclusive) inter‐SU (orthotopic) binding of croton in this positive modulation of the allosteric transitions by a mono‐CBX. Our residue‐dependency analysis shows that the croton PAM effect (on the AA basis, Figure 8a,b) is labile, as much as succin or fumar PAM effects on wt GLIC (see figure 7 in Van Renterghem et al., 2023), since croton PAM effect was fully abolished by any single mutation either in the inter‐SU (orthotopic) pocket, or in the intra‐SU (vestibular) pocket as well. This “all‐or‐none” pattern is consistent with the view that positive modulation by croton requires orthotopic binding, and integrity of the vestibular region corresponding to the intra‐SU pocket. The model proposed for the di‐CBX PAM effect and caffe NAM effect on wt GLIC (see figure 9 in Van Renterghem et al., 2023) now applies also to croton PAM effect on the pre‐β5 variant: binding to the orthotopic site, and involvement of the vestibular region.

It is noticeable that the E181A mutation, when applied on the GLIC AA basis, suppresses croton PAM effect (Figure 8a,b), whereas, applied on a wt GLIC basis, E181A does not suppress croton NAM effect (Figure 5b,d): a mutation in the orthotopic site Loop C suppresses the PAM effect, not the NAM effect, of the same compound. Given that croton is thought to bind exclusively to the orthotopic/inter‐SU site, the data lead to the conclusion that Glu181 is specifically required for (compound‐elicited) positive, not negative modulation.

### 4.5 Unclear mechanism for the mono‐CBX NAM effect

The mechanism is more difficult to understand with the mono‐CBX NAM effect. Indeed, the residue‐dependency in the CBX‐binding pockets is very similar for acet, butyr, and croton NAM effects on wt GLIC (Figures 4 and 5). In all these cases, the “loose” pattern of mutational impact is completely different from the “all‐or‐none” pattern discussed above. With the mono‐CBX NAM effects (Figures 4 and 5), apart for R77A, every mutation has only a weak significant impact, or no impact at all. The greatest impact of removing Arg77 suggests that some coupling between inter‐ and intra‐SU pockets is involved here too, suggesting a necessary inter‐SU binding. However, single mutations in the inter‐SU pocket (R105A) or its orthotopic site entrance (R133A, E177A) have no impact at all (as E181A) or a weak significant impact. Significant mutational impact is observed in the intra‐SU (vestibular) pocket (Tyr102, Glu104), but it is weak too. Here, the model with orthotopic binding and vestibular control is under questioning.

We therefore propose the following interpretation (Figure 9), also supported by crystallographic data: the model with the orthotopic pocket as main binding site still applies, with required integrity of the vestibular pocket. But negative modulation is “easy to reach” and occurs even when binding contacts or residue interactions are poor, whereas positive modulation requires stringent conditions, so that positive modulation is fully lost when a single residue is missing in one of the pockets. This interpretation is supported by the fact that, when croton PAM effect (AA basis) is abolished by any single mutation in the CBX‐binding pockets (“all‐or‐none” pattern), then croton does not become inactive—as were the di‐CBX on most single mutants (wt‐basis; see figure 7 in Van Renterghem et al., 2023). But croton recovers its NAM property (Figure 8a,b), with the NAM‐specific “loose” pattern of residue dependency.

An additional hypothesis may help to explain the mono‐CBX “loose” impact data. In the available structures, acet and propion are found in both inter‐SU (orthotopic) and intra‐SU (vestibular) pockets. And when the inter‐SU (orthotopic) pocket (or its orthotopic site entrance) is not intact, they keep some NAM effect. We hypothesize that intra‐SU (vestibular) binding of saturated mono‐CBX compounds may be sufficient to produce a negative modulation. The vestibule site would then be involved as a secondary, allotopic binding site. In GLIC‐succin co‐crystals, succin is also present in the two pockets. However, succin has no PAM effect at all on the inter‐SU mutants, suggesting that intra‐SU binding alone cannot promote positive modulation. We therefore add to the “in series” model an accessory vestibular (downstream) binding site, putatively involved in negative modulation (Figure 9). Although this point is *compatible* with our data, it is not *demonstrated* by our data.

We finally raise here the hypothesis (Figure 9) that some intracellular acidification may occur following transmembrane permeation of the acid forms of extracellularly applied mono‐CBXs (see Thomas, 1974): a drop in pHi would contribute to negatively modulate GLIC activity (see figure 3 in Van Renterghem et al., 2023). Although we have no data showing the occurrence of a drop in pHi, and although we record using a patch‐pipette filled with a strongly proton‐buffered solution (10 mM HEPES + 10 mM of the tetra‐acido‐basic Ca^2+^‐buffer BAPTA; pH 7.3), we do not exclude some contribution of intracellular acidification to the mono‐CBX NAM effect, which would end up in “loosening” the mutational impact pattern, bringing some “blindness effect” in the analysis of the receptor‐mediated mechanism, in the case of mono‐CBX NAM effects. Indeed, the “slow” recovery from acet inhibition (see current traces in Figures 1b, 2a, 4a) is compatible with the “slow” recovery from pHi lowering. But the consistently “fast” recovery from butyr or croton inhibition (see traces in Figures 2c and 5a,b) excludes a major contribution of intracellular acidification to butyr and croton inhibition. And, finally, the mutational impact pattern for acet (Figure 4), which is not “more loose” than the pattern for butyr or croton NAM effects (Figure 5), but very similar, suggests that the contribution of a pHi effect may be minor with acet as well, comforting a receptor‐mediated mechanism for all mono‐CBX NAM effects.

### 4.6 Impact of R133A on croton NAM effect

Some discrepancy appears between our data and the data published by Alqazzaz et al. (2016), regarding R133A impact on croton effect (for wt GLIC). We found that the quantitative difference in the data has a kinetic explanation: with butyr or croton, inhibition is slowed down in R133A, as visible in our current traces using a protocol with pre‐stimulation (Figure 5a,b). In our protocol with pre‐stimulation, the current in the presence of croton is measured after 20 s of croton action, that is, at the steady‐state of inhibition (on both wt and R133A), leading to the conclusion that R133A has a weak impact on inhibition by croton. In the direct protocol, in wt GLIC, inhibition by butyr or croton occurs during activation by protons (as in Figure 1a), whereas in R133A, delayed inhibition occurs as a decay following the peak of activation by protons, so that measuring the peak current leads to the conclusion that inhibition is almost abolished in R133A. We confirmed this explanation by performing patch‐clamp recordings in the direct protocol, with butyr and croton: then (as Alqazzaz et al., 2016), we found that peak current inhibition is almost fully abolished in R133A, with both butyr and croton. Both protocols are valid; they give slightly different elements of information.

In the crystal structures published in 2020 by Fourati et al., Arg133 (and Glu177) belong to the peripheral entrance of the slightly deeper inter‐subunit CBX‐binding pocket. The authors note that “Arg133 partially obstructs the putative orthosteric pocket.” Here, we propose that Arg133, located in the access corridor (and most probably positively charged), is actually *required* for a fast access of (negatively charged) butyr or croton to their more deeply buried (inter‐SU) binding site. With this explanation, R133A strong impact in the direct protocol (Alqazzaz et al., 2016) and R133A weak impact in the protocol with pre‐stimulation (this report, Figure 5) are both consistent with the relatively long distance between Arg133 and the croton molecule in the crystal structure. The general consistency of the point commented here comforts again the idea that croton and butyr act by binding to the inter‐SU site.

### 4.7 The unexpected impact of N152A

In the GLIC‐CBX co‐crystal structures (Fourati et al., 2020), Asn152 coordinates the second carboxyl group of the di‐CBX molecules bound in the inter‐SU (orthotopic) pocket and does not contact mono‐CBX molecules, [while, at the other end of the di‐CBX molecule, carboxyl group one is in contact with Glu181, Arg105, and Arg77, as with the mono‐CBXs]. We were then expecting that removing Asn152 would abolish di‐CBX effects, and leave mono‐CBX effects unchanged. Indeed, the N152A mutation has *some impact* on the di‐CBX PAM effect (Figure 7c), and *no impact* on the mono‐CBX NAM effects (Figure 7b), consistent with Asn152 *contacting* or *not contacting* the compound, respectively. But succin and fumar PAM effects were unexpectedly *increased* in N152A (Figure 7c). This suggests that Ala substitution of Asn152, or binding of a di‐CBX on Asn152, may result in applying locally common forces involved in promoting positive modulation of the allosteric transitions. This hypothesis is supported by the fact that adding the N152A mutation to the pre‐β5 variant also increased croton (inverted) PAM effect (Figure 8c,d), even though Asn152 does not contact the croton molecule in the available co‐crystal structure. [It may be noted that the facilitating impact of adding N152A to the D86A‐D88A variant is not due to the mild LoF property of the AA‐N152A variant (regarding activation by protons), since the N152A, AA, and AA‐N152A variants show approximately equal ΔpHo_50_ values (Figure 6b)]. From our very unexpected data with N152A and AA‐N152A, we conclude that Asn152, with its local interactions in the protein, exerts in GLIC ECD some resistance to compound‐elicited activation. And that usually accepted interpretations, in a mutational structure–function study, are not always the right ones.

### 4.8 Compound‐elicited activation involves a motion of the pre‐β5 strand

A related conclusion may be derived from our observation that Ala substitution of the double ring of pre‐β5 Asp residues strongly favors a PAM (vs. NAM) effect of compound binding (Figure 7): local interactions of Asp86 and/or Asp88 exert in GLIC ECD some resistance to compound‐driven activation. In contrast, the D86A‐D88A double mutation does not favor low pHo‐controlled activation, as the AA variant is not a gain of function (GoF), but a mild LoF regarding activation by protons (Figure 6b, and Nemecz et al., 2017).

Regarding the position in the agonist (proton) concentration—activation curve, our tests for CBX modulation in the wt or mutants are done at a constant pHo value of 5.0, or proton activity equal to 10^−5^, which is near proton EC_50_ on wt GLIC, and <EC_50_ on GLIC AA (slightly LoF). This may lead to some increased PAM effect on AA versus wt, as predicted by Rubin and Changeux (1966) in the Monod, Wyman & Changeux allosteric model (Monod et al., 1965). But the AA variant and the N152A mutant have equal ΔpHo_50_ values, whereas facilitation of the di‐CBX PAM effect is larger with AA than with N152A (Figure 7c). Moreover, croton and caffe effects are inverted on AA, not on N152A (Figure 7b), showing that something specific occurs with the pre‐β5 variant (vs. N152A), something regarding compound‐driven activation.

### 4.9 The Arg85(*n*)‐Arg77(*n*) and Asp88(*n*)‐Arg77(*n* + 1) ion bridges: Two Arg77 anchoring links are released in CBX‐bound structures

Comparison of the crystal structures for apo‐GLIC (Fourati et al., 2015) on one side, and, on the other side, for GLIC‐acetate (Sauguet et al., 2013) and other GLIC‐CBX complexes (Fourati et al., 2020), leads to remarkable observations regarding Arg77 (at the border between inter‐ and intra‐SU pockets). Arg77 interactions with Arg85 (pre‐β5 residue pointing to the intra‐SU pocket), and with the Asp86‐Asp88 pair (pre‐β5 residues pointing together to the vestibule lumen) are released in CBX‐bound structures.

In an axial view of the pentamer, looking from extracellular to intracellular sides, we number subunits *n* to *n* + 1 clockwise (which is, in a view from the vestibule lumen to the periphery of the pentamer, as in Figure 6a, *n* to *n* + 1 left to right). In the apo‐GLIC structure (4qh5; Fourati et al., 2015), the side chain of the pre‐β5 residue Arg85(*n*) points to the periphery of the pentamer, and, within a subunit, interacts in the middle of the intra‐SU pocket with the side chain of Arg77(*n*) [in its apo‐GLIC orientation]. The pre‐β5 (*n*) next residues, Asp86 and Asp88, have their side chains pointing toward the vestibule lumen, on the (*n*) to (*n* + 1) side of the intra‐SU pocket (*n*) (see Figure 6a). In addition, Asp88(*n*) side chain is involved in an ion bridge with the side chain of Arg77(*n* + 1) [from the adjacent subunit, also in its apo‐GLIC orientation] (Figure 6a). Therefore, Arg77(*n*), in its apo‐GLIC orientation toward the axis of the pentamer, has two anchoring partners: Arg85(*n*) and Asp88(*n* − 1), belonging to the (*n*) and (*n* − 1) pre‐β5 strands, respectively. In all CBX‐bound structures (Fourati et al., 2020; Sauguet et al., 2013), each Arg77(*n*) side chain has pivoted toward the periphery of the pentamer, and coordinates the CBX molecule bound in the inter‐SU pocket(*n*). The side chain of Arg85(*n*) now interacts within the intra‐SU pocket with Glu104(*n*) side chain. And on the axial side of the (*n*) to (*n* + 1) space/interface between two subunits, the Asp88(*n*) to Arg77(*n* + 1) ion bridge is lost (Figure 6a): the side chains of the pre‐β5 Asp86(*n*) and Asp88(*n*) residues point differently in the vestibule lumen. In all GLIC‐CBX co‐structures, the five inter‐SU (orthotopic) pockets are occupied, and the scheme occurs five times: the Asp88(*n* − 1) to Arg77(*n*) bridge is lost, etc.

Therefore, binding of a CBX molecule at the inter‐SU pocket (*n*) [and its coordination by Arg77(*n*)] occurs with, or requires, rupture of the Asp88(*n* − 1)‐Arg77(*n*) ion bridge, [and rupture of the Arg85(*n*)‐Arg77(*n*) intra‐SU interaction]. It may be that inter‐SU CBX binding is facilitated when one of Arg77 anchoring (Asp88, Arg85) in its apo‐GLIC orientation is missing. Consistently, positive modulation is favored in the pre‐β5 variant lacking Asp86 and Asp88.

### 4.10 Release of two pre‐β5 strand anchoring links, and the pLGICs Cys‐Loop (or Pro‐Loop)

Conversely, CBX binding frees the pre‐β5 strand(*n*) from both its Asp88(*n*)‐Arg77(*n* + 1) anchoring to the adjacent subunit, and its Arg85(*n*)‐Arg77(*n*) intra‐subunit anchoring. CBX binding also changes Arg85(*n*) orientation within the intra‐SU pocket, ending in changing the pre‐β5 strand orientation. We suggest that the pre‐β5 strand motion is a major point in coupling CBX binding and pore gating. Given the peculiar properties of the Asp86–Asp88 pair of residues, we chose to use a double mutant to characterize a pre‐β5 involvement. In addition, we have not planned to further analyze the respective contributions of Asp86 versus Asp88 to the amazing properties described for the D86A‐D88A variant.

In GLIC structures, the pre‐β5 strand N‐ter to C‐ter orientation is “ascending,” *that is*, toward the extracellular *apex* of the pentamer. The pre‐β5 and β5 strand is adjacent and antiparallel (“beta sandwich”) to the “descending” β6 strand, which itself is ending in the β6‐β7 Loop, homologous to the “Pro‐Loop/Cys‐Loop” known to be essential to gating in Eukaryote pLGICs. It should be emphasized here that Tyr102 and Glu104, analyzed in our work as intra‐SU pocket residues, belong to this β6 strand descending to the Pro‐Loop/Cys‐Loop. The general location in Eukaryote pLGICs of the pre‐β5‐β5 strand (or β4‐β5 strand) forming Loop Ω, attached to the β5‐β6 “sandwich” ending with the Pro‐Loop/Cys‐Loop, suggests that our mechanistic model, with inter‐SU binding, and vestibular control of a pre‐β5‐β5 strand motion also dragging the Pro‐Loop/Cys‐Loop, may have some relevance to the mechanism of gating control by neurotransmitters in human pLGICs.

### 4.11 Relevance of the model to Eukaryote pLGICs

Some authors questioned the presence in the ECD of Eukaryote pLGICs of a cavity homologous to the prokaryote vestibular pocket, as this may end up in a new allotopic target site for NAM or PAM therapeutic compounds. Is it possible to awake a functional vestibular site in human pLGICs? The presence in type 3 serotonin receptors (5HT_3_Rs) of an empty cavity at this location was noticed by Hu et al. (2018), who also defined the β4‐β5 loop as Loop Ω. In addition, the point was analyzed systematically in Eukaryote pLGICs by Brams et al. (2020). They concluded that, as in 5HT_3A_ receptors, a vestibule cavity is accessible in several cationic pLGIC subunits, among which muscular type nicotinic receptor subunits, due to a “Ω‐open” conformation of the loop. In other cationic pLGICs subunits however, including (non‐α7) neuronal nicotinic subunits, the vestibule site entrance is obstructed by its own Loop Ω in a “Ω‐in” conformation, and therefore non‐accessible to ligands. Brams et al. also showed that MTSEA‐biotin modification of engineered Cys mutants in the vestibule site (in particular with Leu151 in the β6 strand) produces a PAM effect on the serotonin‐activated 5HT_3A_ receptor, showing that it is possible to target the vestibule site for modulation of the 5HT_3A_ receptor, and putatively other Eukaryote pLGICs.

In anionic pLGICs, Loop Ω (“Ω out,” Brams et al., 2020) protrudes from subunit (*n*) (complementary side) into a cavity of the neighbor (*n* + 1) subunit (principal side), on the axial/vestibule/lumen side of the ECD. This cavity (*n* + 1), occupied by the neighbor subunit Loop Ω (*n*), corresponds to the prokaryote vestibular pocket, and is therefore adjacent to the orthotopic, neurotransmitter binding site. In GLIC, fumar or croton binding, associated with Arg77 pivot rotation to the CBX‐bound orthotopic/inter‐SU site, releases the Asp88(*n*)‐Arg77(*n* + 1) ion bridge [the pre‐β5 strand (*n*)‐(*n* + 1) anchoring], and frees Arg85 (the pre‐β5 strand) from its intra‐SU anchoring through other intra‐SU residues. It may be hypothesized that the Asp88(*n*)‐Arg77(*n* + 1) ion bridge evolved toward the insertion of a whole protruding loop in a pocket within the neighbor subunit. It may be that the ancestral 2‐arm system controlling the position of the pre‐β5 strand (and β5‐β6 sandwich, and Pro‐Loop) from the orthotopic site in GLIC was replaced during evolution by a modern “joystick” (Loop Ω), hold in hand by a vestibular pocket, itself manipulated by the adjacent neurotransmitter binding site, a “joystick” which drags the β5‐β6 sandwich ending with the Cys‐Loop.

## 5 CONCLUSION

The electrophysiological studies presented in the present report and in Van Renterghem et al. (2023), and previous crystallographic studies from Sauguet et al. (2013) and Fourati et al. (2015, 2020), together establish that the orthotopic site (inter‐subunit site), the future neurotransmitter binding site, is functional in GLIC, the ancestor pLGIC from a bacterial species with pre‐cambrian characteristics. GLIC orthotopic site is involved as a functional binding site in positive modulation of low‐pHo activated GLIC, in both wt (fumarate, succinate) and D86A‐D88A (crotonate), without conferring agonist properties. The orthotopic agonist site in Eukaryotes may derive from an orthotopic site with no agonist property, but early involved in positive modulation of pH‐controlled allosteric transitions.

Binding at the orthotopic site is most probably involved as well in negative modulation by caffeate, crotonate, and butyrate, which all give “fast” effects and “fast” reversibility (seconds). Whereas “slow” components of negative modulation by acetate or propionate (with “very slow” reversibility, 3–10 min) may involve a drop in intracellular pH, also shown to negatively modulate GLIC.

In this complex system, with two binding sites (orthotopic/vestibular), two types of effect (PAM/NAM), and two types of compounds (di‐/mono‐CBX), we exclude a model with one site/one effect. We propose that a main binding site (orthotopic) and the vestibular region are involved “in series” in both PAM and NAM effects, with or without vestibular binding on top.

Orthotopic and vestibular sites are in all cases equally impacted by mutations, with an “all‐or‐none” pattern (two sites) for PAM effects and the high affinity NAM effect of caffeate, and a “loose” pattern (two sites) for low‐affinity NAM effects. Therefore, the strongest arguments supporting the orthotopic site as the main binding site come from GLIC‐CBX co‐crystal structures. (1) The CBX is found in the orthotopic site in all co‐structures available, when only acetate, propionate, and succinate occupy also the vestibular site. (2) The pivot movement of Arg77 from the empty vestibular site toward the CBX into the orthotopic site occurs in all the co‐structures. It is only with the hypothesis of an “in series” mechanism, with the orthotopic site as main binding site, that the whole set of structural and functional data finally becomes consistent.

## AUTHOR CONTRIBUTIONS

CVR set up patch‐clamp, prepared some mutants used in Figures 7 and 8, designed and performed patch‐clamp experiments (including cell preparation), analyzed data (including statistical treatment), prepared patch‐clamp figures, and performed part of *Xenopu*s oocyte electrophysiology. ÁN performed part of *Xenopus* oocyte electrophysiology and prepared Figure 6a and Figures S1 and S2. KM and CVR performed molecular biology constructs and production. P‐JC initiated and supervised the work. CVR wrote the first version of the article. All authors contributed to revising the article. All authors have approved the final version of the manuscript and agree to be accountable for all aspects of the work. All persons designated as authors qualify for authorship, and all those who qualify for authorship are listed.

## FUNDING INFORMATION

ÁN was supported by the Agence nationale de la recherche (ANR grant 13‐BSV8‐0020 “Pentagate”). CVR and P‐JC have permanent CNRS positions, KM has a permanent Institut Pasteur position. P‐JC's laboratory belongs to IP, CNRS, and UPC, supported by public funding and donators.

## CONFLICT OF INTEREST STATEMENT

The authors declare no competing interest.

## ETHICS STATEMENT

An immortalized cell line in culture was used for patch‐clamp recordings. Commercially available oocytes were used in two‐electrode voltage clamp recordings. The company providing defolliculated primary oocytes from *Xenopus laevis* surgery is Certified by Ministry of Nature, Environment and Consumer Protection, Germany.

## Supporting information


Figure S1.

Figure S2.

## Data Availability

For patch‐clamp electrophysiology data, representative raw current traces (current recorded versus time) are available in most of figures (Figures 1, 2, 3, 4, 5, 7, 8; not in Figure 6). For electrophysiology data presented as bar graphs, individual value data points (one per cell in each condition) are superimposed to mean ± SD (Figures 3b, 4b, 5c,d, 6b, 7b,c, 8b,c), and the number of cells tested is indicated in brackets in each bar graph category name. Mean ± SD is represented in Graphs for [concentration] – [current] “dose–response” curves (Figure 2b,d). The number of cells included is then indicated in the figure legend and/or text. In all cases, “*n*” includes cells from at least two DNA transfections (tk‐ts13 cells) or two DNA injections (*X*. oocytes). A statistical comparison of pairs of samples was performed in experiments with carboxylates on GLIC mutants (Figures 4b, 5c,d, 6b, 7b,c, 8b,c). *p* Values are then indicated near bar graphs in the figures, and most of the time also in text.
