# Supplementary figures and images for: Short‐chain mono‐carboxylates as negative modulators of allosteric transitions in Gloeobacter violaceus ligand‐gated ion channel, and impact of a pre‐β5 strand (Loop Ω) double mutation on crotonate, not butyrate effect
Source: Physiol Rep. 2024 Feb 11;12(3):e15916. doi: 10.14814/phy2.15916 (PMC10859675; doi:10.14814/phy2.15916)

*A*

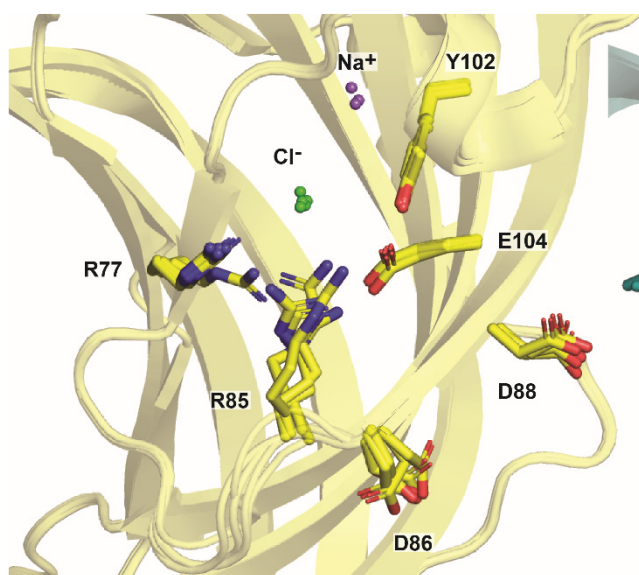

*B*

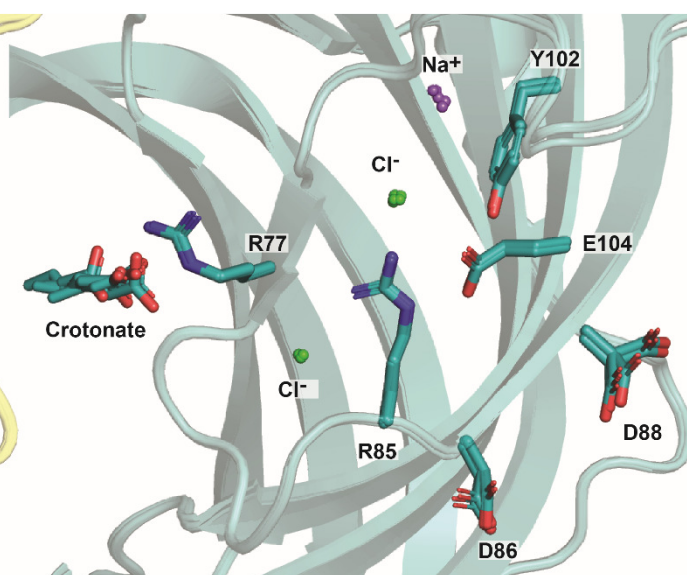

Supplement: Supplementary file 1 — Figure S1. [file PHY2-12-e15916-s002.pdf]

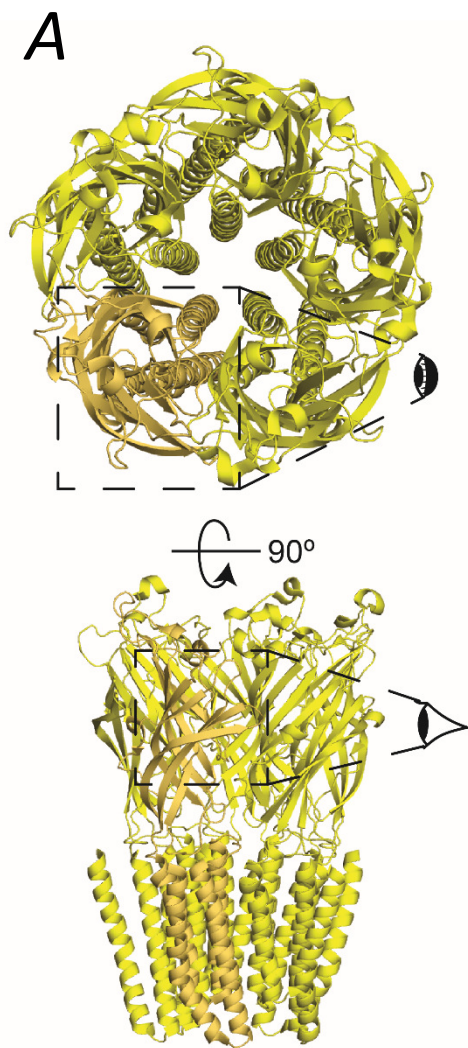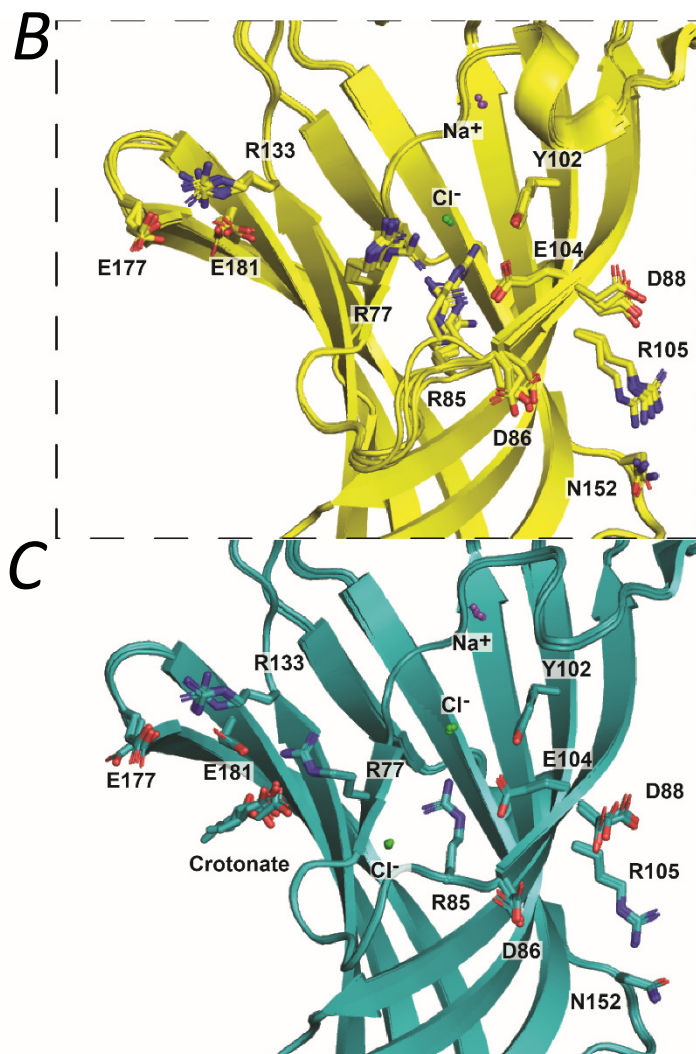

Supplement: Supplementary file 2 — Figure S2. [file PHY2-12-e15916-s001.pdf]
